# A content analysis of e-cigarette marketing on social media: Findings from the Tobacco Enforcement and Reporting Movement (TERM) in India, Indonesia and Mexico

**DOI:** 10.3389/fpubh.2022.1012727

**Published:** 2022-11-08

**Authors:** Nandita Murukutla, Melina S. Magsumbol, Hana Raskin, Sharan Kuganesan, Silvia Dini, Carlos Martinez-Mejia, Rachfiansyah, Benjamin Gonzalez Rubio Aguilar

**Affiliations:** Policy Advocacy and Communication Division, Vital Strategies, New York, NY, United States

**Keywords:** e-cigarette marketing, social media, electronic nicotine delivery systems (ENDS), FCTC, digital monitoring

## Abstract

**Background:**

The use of e-cigarettes is proliferating globally, especially among youth and even children. Marketing is a known risk factor for e-cigarette initiation, yet little is known of e-cigarette marketing on social media in low- and middle-income countries. This study compares e-cigarette social media marketing in India, Indonesia, and Mexico, three such countries with different regulatory environments.

**Methods:**

Instances of e-cigarette marketing on social media platforms were identified via the Tobacco Enforcement and Reporting Movement (TERM), a digital tobacco marketing monitoring system. Through systematic keyword-based searches, all tobacco marketing posts observed between 15 December 2021 and 16 March 2022 were included in the analysis. The final sample included 1,437 e-cigarette-related posts on Instagram, Facebook, Twitter, YouTube, and TikTok, which were systematically content analyzed by independent coders after inter-reliability (Cohen's Kappa K > 0.79) was established using a theory-derived codebook. The final data is represented in percentages and frequencies for ease of presentation.

**Results:**

We observed e-cigarette marketing online in all countries studied, yet there was variation in the volume of marketing and types of accounts identified. In India, where e-cigarettes were comprehensively banned, we identified 90 (6%) posts; in Mexico, where e-cigarettes were partially restricted, 318 (22%) posts were observed; and in Indonesia, where there were no restrictions, 1,029 (72%) posts were observed. In both India and Mexico, marketing originated from retailer accounts (100%), whereas in Indonesia, it was primarily product brand accounts (86%). Across countries, e-cigarettes were mostly marketed directly to sell products (India: 99%, Indonesia: 69% and Mexico: 93%), though the sales channels varied. Product features, including e-liquid flavors, device colors and technical specifications, was the most prominent message framing (India: 86%; Mexico: 73%; Indonesia: 58%). Harm reduction messaging was most popular in Mexico (8%) and was not common in Indonesia (0.3%) or India (0%).

**Conclusion:**

Our study provides important insights for tobacco control stakeholders on the evolving nature of e-cigarette marketing in low- and middle-income countries. It underscores the presence of e-cigarette marketing, including in countries where comprehensive regulations exist, and suggests the importance of continuous monitoring to keep up with industry practices and strengthen tobacco control stakeholder efforts to counter them.

## Introduction

The use of e-cigarettes is proliferating globally, especially among youth and even children (1–3). This is concerning because while the public health benefits of e-cigarettes as a cessation aid are debated, there is mounting evidence of their harm: A recent review of global research on e-cigarettes found conclusive evidence that e-cigarettes can cause respiratory disease and other adverse health outcomes, and that they serve as a “gateway” product, increasing the likelihood of never-smokers—especially youth—using combustible tobacco products (4).

Marketing is a key risk factor for e-cigarette initiation (5, 6). Exposure to e-cigarette marketing is associated with experimentation and current use of e-cigarettes among youth and children, as well as increased susceptibility of use among those who have never used e-cigarettes or combustible cigarettes (7–9). Marketing is also a key channel by which industries seek to influence the regulatory environment affecting them (10). In the case of the e-cigarette industry, this has entailed presenting e-cigarettes as a harm reduction device—a tool necessary for addicted smokers to gradually quit tobacco use—and therefore a product that should not be tightly regulated (11).

Globally, government responses to the rise of e-cigarettes and the risks they pose vary (12). As of 2021, 79 countries have adopted one or more legislative measures to regulate e-cigarettes; 32 countries have banned e-cigarette sales, while 22 have banned the advertising, promotion and sponsorship of e-cigarette devices, e-liquids or both (3). However, the internet has posed a significant challenge to the implementation of these policies (12).

### E-cigarette marketing on social media

Digital media is a core part of the e-cigarette industry's strategy to promote products and its industry (13, 14). Social media, in particular, allows for products to be marketed to young people across borders at low cost and under less oversight than traditional media platforms. Social media also allows for the direct integration of e-commerce platforms and for more direct, peer-like interaction with users—a strategy that tends to attract youth (3, 15, 16). Research from the United States, Australia and other high-income countries has shown that e-cigarette marketing is prevalent on social media platforms such as Instagram, Facebook, YouTube, and Twitter (17–21), and that it is associated with youth uptake of e-cigarettes (5, 8).

However, there is less known about online e-cigarette marketing in low- and middle-income countries with large online youth populations (22). This is of particular concern because of the large youth populations in countries that have been the target of e-cigarette industry ambitions (23). Emerging evidence suggests the prevalence of online e-cigarette marketing: A recent report on Indonesia, which has a nascent, fast-growing e-cigarette market (24) and a large population of youth active online (25), found that e-cigarette marketing was prevalent on social media platforms and used a range of youth-oriented tactics to promote the use of e-cigarettes and their components (26).

Moreover, there is a need for rapid, continuous data that can track the evolving practices of the tobacco and e-cigarette industry in a rapidly evolving digital environment (22). Marketing no longer consists of simply direct advertising, but also includes more covert tactics such as: event sponsorships; “corporate social responsibility” activities; and the use of less easily traceable front groups, celebrities and social media influencers (20, 27). Continuous monitoring of online e-cigarette marketing is crucial to the successful introduction and implementation of marketing laws and enforcement of violations, particularly online where it is easier for industry players to avoid oversight (22, 28).

To address this need, Vital Strategies developed a digital media monitoring system, the Tobacco Enforcement and Reporting Movement (TERM) (29) that continuously tracks online tobacco marketing in India, Indonesia and Mexico—three geographically diverse and regionally influential countries with large populations of youth online. Evidence generated by TERM is shared regularly with stakeholders in each country, including ministries of health, tobacco control officers, academics and journalists.

The Asian region is home to more than half of the world's young people, many of whom are regularly using the internet and social media, which puts them at increased risk of exposure to tobacco and e-cigarette marketing (30). In India, in 2019, two-thirds of 504 million active internet users were between 12 and 29 years old, and in Indonesia, 44% of internet users or 118 million people are between 5 and 34, with nearly 18% between the ages of 5 and 18 (31, 32). Recent surveys in India and Indonesia have shown that youth are in fact being exposed to tobacco and e-cigarette marketing on social media (33–35). Mexico also has a large population of young people online: approximately 40% of online users are between ages 6 and 24, corresponding to 34.9 million people (36).

Data from the most recent Global Adult Tobacco Surveys in each country show that e-cigarette use among people ages 15 and above ranges from 0.02% in India to 0.6% in Mexico to 3% in Indonesia (37–39). Other studies in Indonesia have found that e-cigarette use among youth ranges from 10.7% in the city of Yogyakarta to 11.8% in Jakarta (40, 41).

At the time of this study, e-cigarette regulations across the countries also differed: In India e-cigarettes were comprehensively banned, including their importation, distribution, advertising and sale (42). In Mexico, midway through the study, the national tobacco control law was revised to ban the trade, sale, distribution, exhibition and promotion of any product that resembles a tobacco product, which was intended to apply to e-cigarettes but left room for loopholes (43). Prior to that only the importation of e-cigarettes was banned (44). Following the culmination of this study, e-cigarettes were banned outright (45). In Indonesia, there was no national law restricting the sale, use or advertising of e-cigarettes (46).

This study draws from TERM to fill the gap in knowledge on the extent and type of e-cigarette marketing prevalent on social media in these three countries. In addition, the varying policies on e-cigarettes provides an opportunity for assessing the impact of regulatory environment on online e-cigarette marketing.

## Methods

This study was conducted in three countries—India, Indonesia and Mexico—that are currently monitored by TERM, a digital media monitoring system that records instances of online tobacco marketing on social media and news sites. TERM uses a parsimonious and systematic research approach to facilitate the rapid generation of evidence for policy implementation and decision support (47). TERM was designed and is implemented by Vital Strategies with technical inputs from tobacco control experts in India, Indonesia and Mexico. Data collection and preliminary analysis is conducted by Radarr (48), a social and digital data analytics company that uses AI and machine learning software to track publicly available posts on digital platforms. For this study, only content from social media was included in the analysis.

### Data collection

We analyzed all publicly available posts related to e-cigarettes on social media identified via TERM over a 3-month period between 15 December 2021 and 16 March 2022. This data was extracted from routine TERM monitoring in the three countries, which captured instances of tobacco marketing for conventional and newer tobacco and nicotine products, including e-cigarettes, on social media by tracking the accounts of all known tobacco companies and brand products on the social media platforms monitored by TERM. These platforms were Instagram, Facebook, YouTube, TikTok, and Twitter, with the exception of TikTok in India where it was banned.

The list of accounts for analysis were identified purposively and through exploration of online content in a multi-step process. During the first step, a list of prominent tobacco companies and product brands that were sold in each country were identified through a thorough process of consultations with tobacco control experts in each country and by reviewing Euromonitor market share reports. After obtaining a comprehensive list, active social media accounts were identified and configured in the Radarr platform for tracking. Next, additional accounts were identified by systematically searching social media content with keywords. The keywords to identify instances of online tobacco and e-cigarette marketing were systematically curated based on expert inputs and by reviewing relevant literature (49–53). Keyword-based Boolean searches, where keywords and hashtags, such as “e-cigarette” and “vape” were stitched together using special operators (“and,” “or”) with company or brand names (“Geekvape” or “#Geekvape”) were used to identify marketing instances. The Boolean search strategy was found to be particularly important for the identification of new accounts, and for newer tobacco and nicotine products, such as e-cigarettes, which had more diffused brand presences. A sample of keywords used for Boolean searches in each country is presented in Appendix 3.

We included all publicly accessible, organic marketing posts from configured accounts, and posts from accounts discovered using keywords that directly or indirectly promoted conventional tobacco or new nicotine and tobacco products within the study timeframe (for details see Appendix 1). Only posts that were written in English and in the commonly spoken languages of each country (Hindi, Indonesian and Spanish) were included. Each post consisted of an image or a video, which may have been accompanied by text, hashtags or emoticons. The text accompanying each post was translated to English using Google Translate on Chrome.

The content created by influencers that was shared or cross-posted by tobacco companies or product brand accounts was included in our dataset. However, posts originating from the personal social media accounts of influencers, brand ambassadors and journalists were excluded from the dataset since it was unclear if they reflected the official position of the tobacco companies and because it was difficult to discern financial or material relationships with the product brands, which was a criterion for inclusion in our study.

A total of 6,337 instances of online tobacco marketing were identified in the study period. Of those, 1,437 posts (23%) about e-cigarettes were extracted for analysis in this paper.

### Data coding

A standardized codebook was developed based on the published literature (49–53), expert inputs and initial data exploration. The full codebook is available in Supplementary Appendix 1. The following are the key analyses conducted on the posts that are reported in this paper:

- Product type: We categorized all posts based on the type of product marketed, such as: smoking tobacco, which included indigenous products like bidis in India and kreteks in Indonesia; smokeless tobacco; allied products (e.g., surrogate products or those non-tobacco products to which the company or product brand has been extended); e-cigarettes; and other newer products, including heated tobacco products and nicotine pouches. While TERM at large analyzes marketing of allied products, this category was not applicable to this analysis of e-cigarette marketing (for more details see Supplementary Appendix 1: Codebook).
- Account type: This entailed designating the social media account as created by tobacco or nicotine product brands, company brands, third-party retailers or product/company-affiliated community groups.
- Product brand: Name of promoted product.
- Company: Name of the parent company.
- Country of origin: Country where the parent company was located.
- Platform: Social media platforms, including Facebook, Instagram, Tik Tok, etc.
- Engagement: Sum of audience engagement including number of likes/loves, comments/replies and shares.
- Marketing tactics: The type of marketing strategy used, which captured the intention of the marketer, such as selling products or creating brand endearment and loyalty.
- Message framing: Underlying theme around which the whole message of the post was built.

The Radarr digital platform was programmed to code the content according to the codebook. Trained human coders, including three authors to this paper (SD, SK, CM-M), then checked the coding to ensure its accuracy. They also manually conducted the content analysis for some of the more subjective variables according to the coding framework. Following careful discussion, particularly with study lead (NM) and second author (MM), the coders were trained to use the coding framework and independently analyzed 10% of the dataset for the two categories that were deemed to be the most complex and open to subjective interpretation: marketing tactics and message framing (Table 1). Once inter-rater reliability of Cohen's kappa (K > 0.79) was established in coding of 10% of posts on the variables “marketing tactics” and “message framing,” the coders then went on to code the remaining content independently. Any discrepancies were discussed with the study lead and resolved by the study team.

**Table 1:** Definition of marketing tactics and message framing used in the analysis for this paper.

**Variable**		**Description**
Marketing tactic	Direct advertising	Promotes the sale or use of tobacco and new nicotine and tobacco products, such as e-cigarettes and heated tobacco products, in a general way.
	Price promotions	Promotes the sale or use of tobacco products through price discounting.
	Events, occasions, sponsorships	Pairs the tobacco product brand with events, occasions, sponsorships and contests. Any publicized events, occasions or contests, used for promoting tobacco companies, including “any form of contribution,” financial or otherwise.
	Corporate social responsibility	Pairs the company/brand or product with socially responsible activities (e.g., water conservation projects, financing vaccination drives)
	General profile raising	Intended to raise the profile of the brand/company but does not fall into any of the other categories.
Message framing	Community celebrations and festivals	Posts commemorating a specific event or community celebration. This category also includes posts commemorating birthdays or deaths of famous people including religious figures and politicians.
	Entertainment	Posts that promote the product as being entertaining and fun. This category also includes posts that use entertainment-related content including memes, funny videos or jokes.
	Environment eco-awareness	Any post that addresses climate change, conservation, sustainable development goals, or support for environment/conservation.
	Glamorization	Posts that associate use of products as aspirational, luxurious or part of an ideal, fashionable lifestyle.
	Health claims	Posts that present the product as healthier than other products or as less harmful than conventional tobacco products.
	Informational	Posts that instruct viewers how to use a product. This category also includes posts that provide background information on the company.
	Personal care and wellness	Posts that associate products with relaxation or stress management. This category also includes posts that frame products as being used for social bonding, including forming community around use of the product with like-minded peers.
	Product features	Posts that primarily emphasize the available choices of product flavors and design including device colors, as well as technical specifications of the product. Posts without any text descriptions or keywords that only display the product are also included in this category.
	Social welfare	Posts that showcase activities sponsored or supported by the company/brand that are meant to improve their public image. Any posts associating the company with social welfare schemes, livelihood initiatives, women's empowerment, entrepreneurship, educational scholarships, etc.

### Data analysis

We used Microsoft Excel for Mac (v.16.6.1) and Stata/Standard Edition for Mac (v.17.0) to conduct descriptive analyses of the types of tobacco or nicotine products marketed, social media platforms used, social media accounts, and marketing tactics and message framing used.

We conducted a bivariate analysis of the distribution of one variable across the categories of a second variable through cross-tabulation. Specifically, we described social media platforms on which companies made accounts, engagement metrics based on different types of message framing, and e-cigarette marketing by types of accounts across social media platforms. In addition, we analyzed engagement by each type of message framing and social media platform. We also described the product brands and parent companies observed in each country. Examples of each marketing tactic and message frame were provided for each country (Tables 2, 3).

**Table 2:** Examples of e-cigarette marketing tactics observed in India, Indonesia, and Mexico.

**Marketing Tactic | Country**	**India**	**Indonesia**	**Mexico**
Price promotion	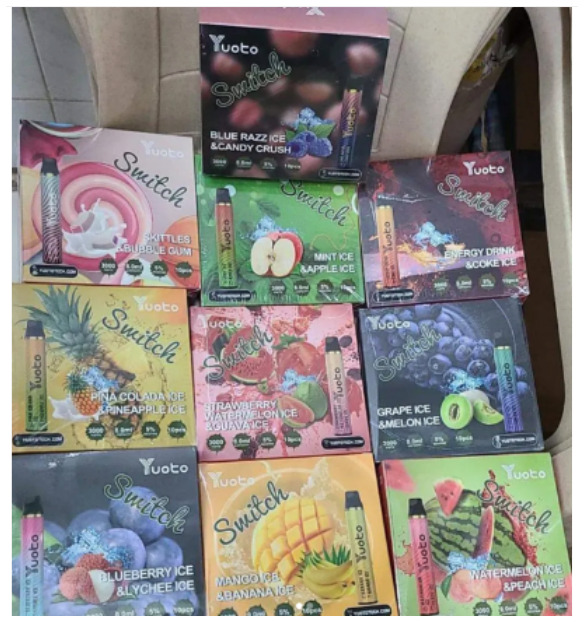 Source: Vape Bar India Instagram page https://www.instagram.com/p/CZjtS_1PGWg/	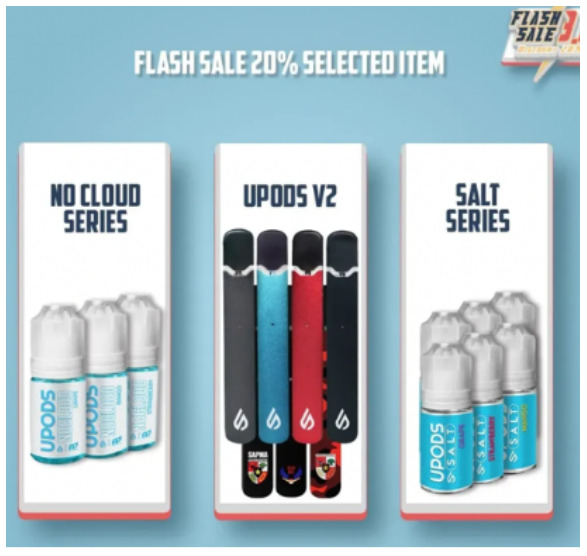 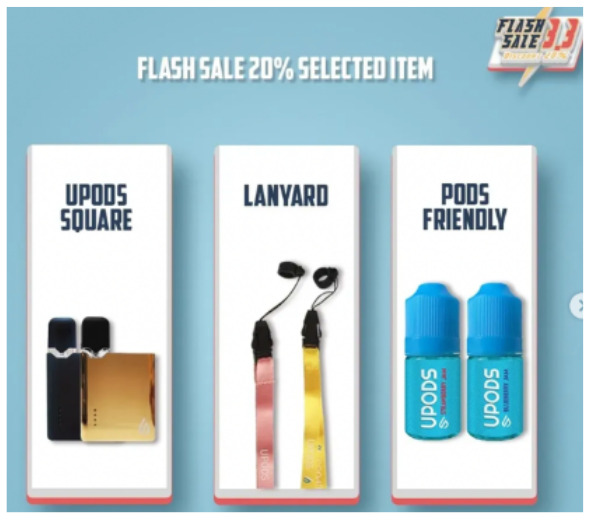 Source: Upods_id Instagram page https://www.instagram.com/p/CamctgaPLnb/	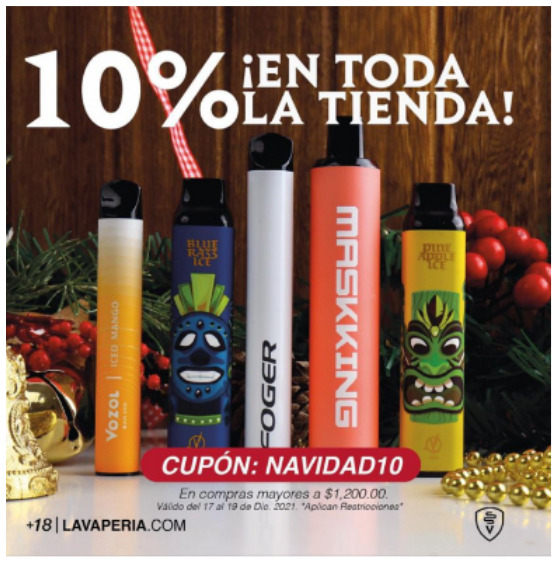 Source: La Vaperia Vape Shop Facebook page https://www.facebook.com/660288400780252/posts/2314899645319111/
Caption translation	Get 10 pcs and get 3 free. Offer valid till 14 february. Get your vapes now	Flash Sale 3.3 !!! Upods Again Ngadin Flash Sale 3.3 Lho. Discount 20% for certain products… # Switchit # switchgang # oleapapeupods # everyonecanswitch	LAST HOURS! 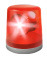 Do not stay without your Christmas present 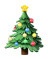 .10% discount throughout the store 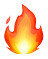 making an equal to or greater than $ 1,200.00 pesos. Use the coupon: Christmas10 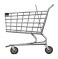 … #vapermexican #Instavape #itsjustvapor #Vapeators #vapelove
Direct advertising	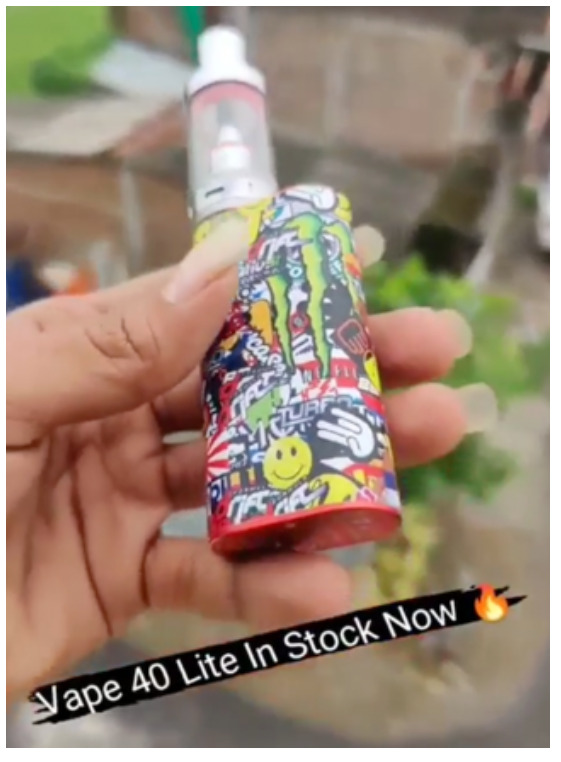 Source: vape_wholesaler_india Instagram page https://www.instagram.com/p/CYVu7FxpJsd/	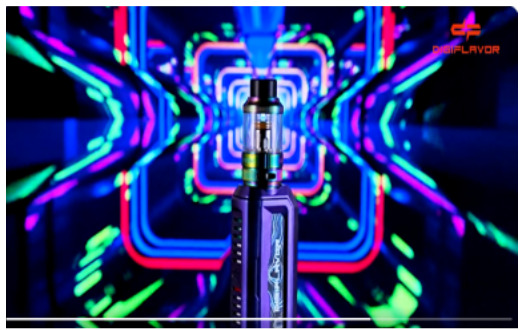 Source: GeekVape Twitter account https://twitter.com/GeekvapeTech/status/1480447946831581186	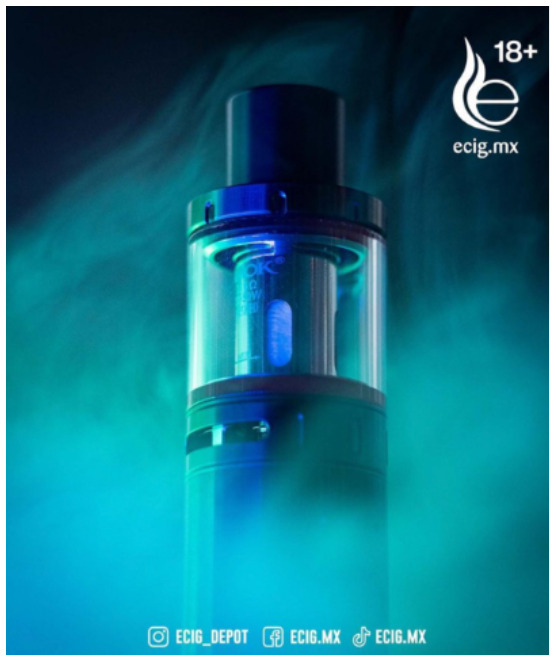 Source: ecig.mx Facebook page https://www.facebook.com/720882044675543/posts/4689966671100374/
Caption translation	No text	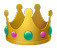 DF XP 77W Kit 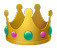 - The Crown Has Arrived! 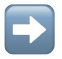 Multi-Use Digi-Crown Customizable 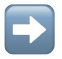 LED Effects 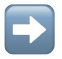 Safety Protection Switch 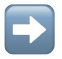 4.5 ml XP Pod Tank… #geek #GEKUP #Digiflavor # XP77 #Crown Not for U.S. Market. https://t.co/u72pncurip.	The Vape Pen 22, a classic in starter kits, now in its new and improved version. Come for yours and finally stop the habit of smoking. #Smok #vapefriends #vapeworld #vapearnoesfumar #vapestyle #handcheck #elvapeosalvavidas 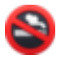 #vapenations #vapecommunity #vapefamily #elvapeonoestabaco
Events, occasions, sponsorships	NA	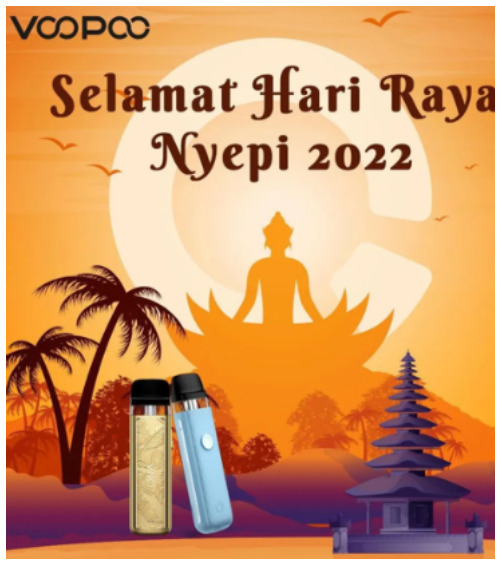 Source: voopoo_indonesia Instagram page https://www.instagram.com/p/CaoRDj0pwe7/	NA
Caption translation		In the midst of the silence of the Nyepi holiday, make the moment a moment for introspection so that peace always accompanies us. Happy Nyepi New Year Saka 1944. # Harinyepi2022	
General profile raising	NA	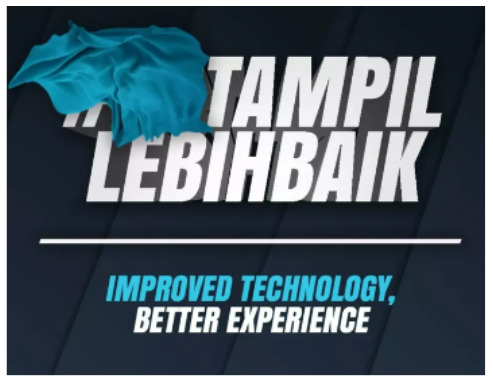 Source: Upods_id Instagram page https://www.instagram.com/p/CY1CKL5P0H_/	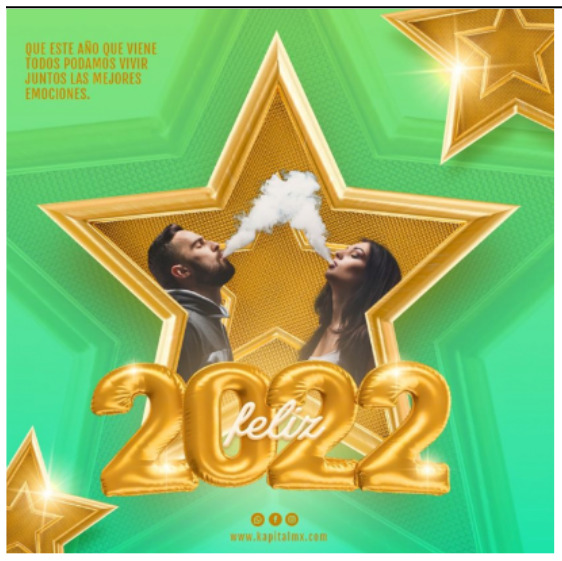 Source: Kapital Smoke & Vapor Facebook page https://www.facebook.com/669235996458669/posts/4520081384707425/
			
Caption translation		Let's Having a Better Experience Than Ever With Us Switch Gang!.. # Switcit # Switchgang # Kitterapeupods #upodsIndonesia	Goodbye 2021. Hello 2022! 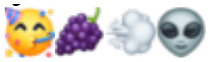 That this coming year we can all live together the best emotions. #vapes
			#vapestagram #mexico #cdmx #love #art #style #travel #followme #new*year* #smile #music #girl #vapes #vapestagram Exclusive product for of age. This product contains nicotine, an addictive substance. Do not share content with minors.

**Table 3:** Types and examples of message framing used in India, Indonesia, and Mexico.

**Message frame | Country**	**India**	**Indonesia**	**Mexico**
Product features	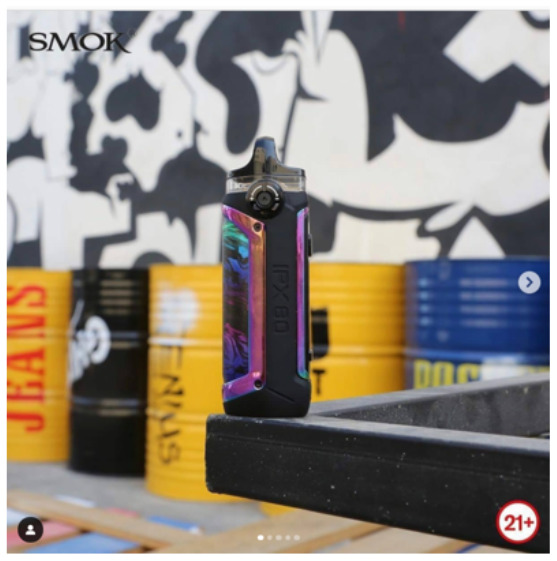 Source: indian_vape_shop Instagram page https://www.instagram.com/p/CYoBRVYJM4Y/	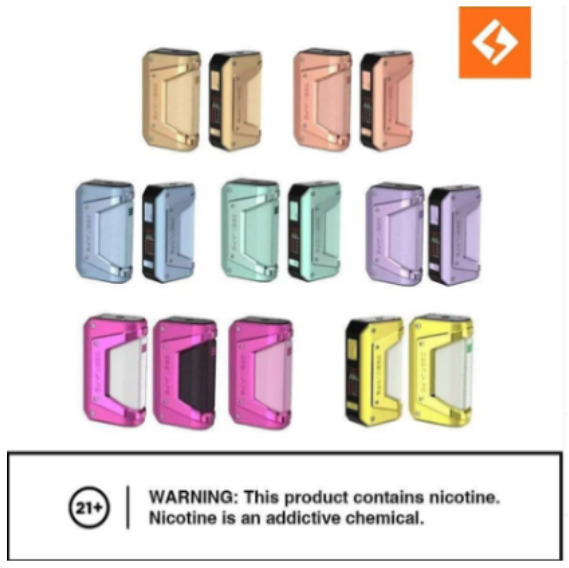 Source: geekvape.indonesia Instagram page https://www.instagram.com/p/CYLq50lviOx/	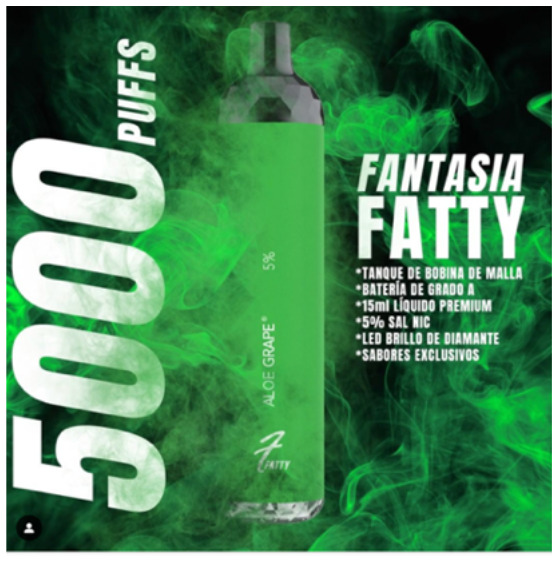 Source: kapitalsmokeandvapor Instagram page https://www.instagram.com/p/CailIQLhvN3/
Caption translation	#SMOK #IPX80 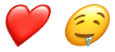 . ✓IPX67 protection ✓80 watt ✓3000mah battery ✓0.96-inch display. WARNING: This product is intended to be used with e-liquids that may contain nicotine. Nicotine is an addictive chemical. For adult use only. #smoktech #smokipx80 #vape #clouds #smoking #ecig #ecigs #vapes #vapekit #vapekits #vapersofinstagram #vaper #vapefeed #vapegirl #vapegram #vapegirls #vapelove #vapelife #vapefam #vapegear #vapebox #vapefamous #vapefamily #vapenation #vapestagram #vapeon #vapestuff #vaporizers	What if Mimin says, “NEW YEAR NEW COLOR” Maybe it's not the same as the one in the photo, but are you ready for the new L200 color? 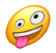 Mimin already has 5 colors	It is manufactured with first quality ingredients, includes a mesh resistance that offer maximum flavor, with a high steam output, has a rechargeable grade battery, with 15 ml of premium liquid and 5% nicotine salts. # Fantasy # vapes #vapestagram #mexico #cdmx #love #Art #style #travel #followme #fitness #beautiful #vapes #vapestagram... Exclusive product for those of legal age. This product contains nicotine, addictive substance. Do not share content with minors # fantasia
Entertainment	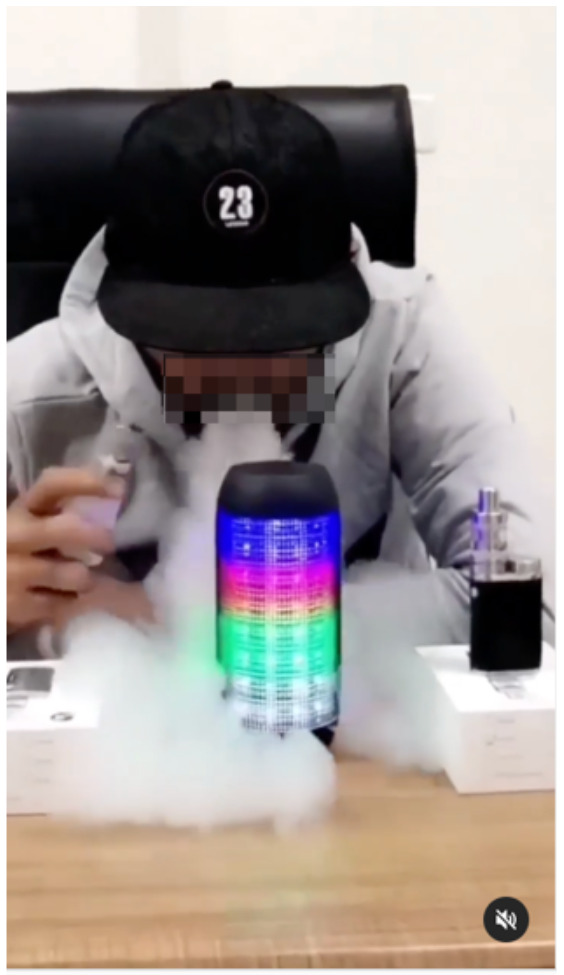 Source: vapers_stop_india Instagram page https://www.instagram.com/p/CZJsWCxl9yq/	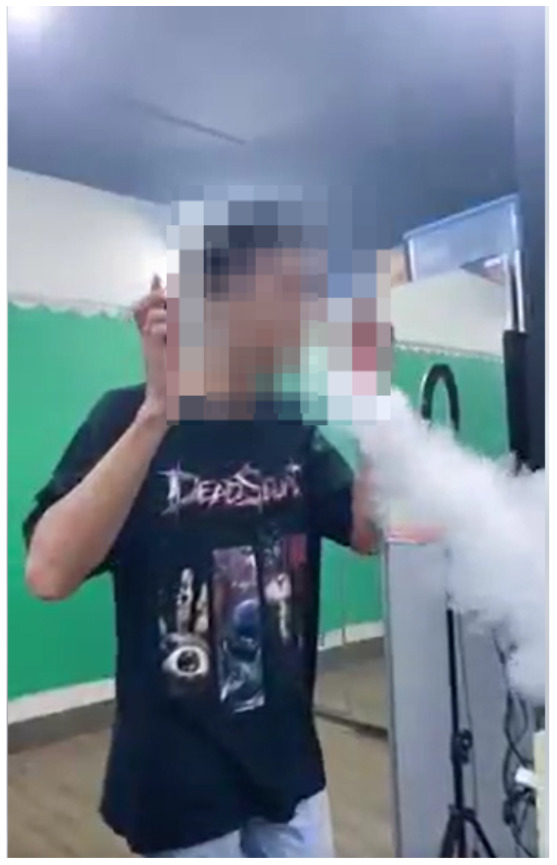 Source: GeekVape Indonesia Facebook page https://www.facebook.com/105583304120083/posts/631142141564194/	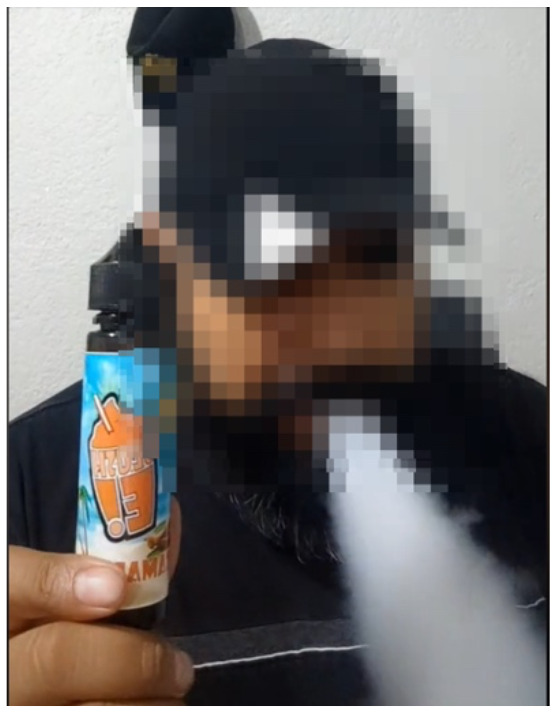 Source: lasoberbiastore TikTok page https://www.tiktok.com/@lasoberbiastore/video/7066684557902253318
Caption translation	#reels #instagram #reelsinstagram #trending #love #viral #explore #instagood #explorepage #tiktok #reelitfeelit #india #follow #instadaily #photography #reel #followforfollowback #likeforlikes #fyp #like #memes #foryou #reelsvideo #fashion #music #reelkarofeelkaro #reelsindia #instagramreels #ke #bhfyp	After being observed 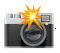 vapes_aby turned out to be tricky, it could be like it. What's because it uses a 65FC obelisk so yes	Slush Tamarind !!!!!!! #dinoterraza #eciquid #vapeordie #lasoberbiastore #cirality #Vapeo #Vapearessalud
Personal care and wellness	N/A	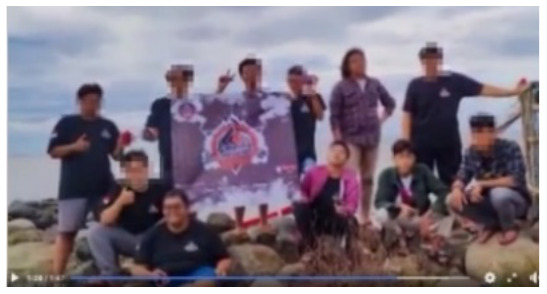 Source: GeekVape Indonesia Facebook page https://www.facebook.com/GeekVape.Indonesia/videos/setelah-sekian-lama-kami-lalui-kami-temukan-tim-yang-solid-persaudaraan-pemuda-k/1253690881782732/	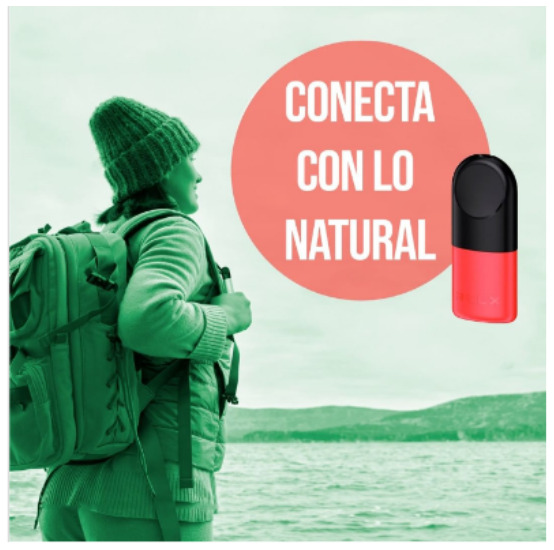 Source: kapitalsmokeandvapor Instagram page https://www.instagram.com/p/CY7lZXor3mj/
Caption translation	N/A	After all this time we went through, we found: - a team of solid-brotherhood - creative youth and became an extraordinary community. Instead. We will do something extraordinary, something new, something to remember !!! So, don't miss it. Make sure you monitor every latest info from us! #Geekvape #geekvapeindonesia #Geekvapetech #probolinggo #vapecommunity #vapenation	In this 2022, as you, we want to break with the routine. With the Relx Pods, there is a true change of air. # Vapes #vapestagram #mexico #cdmx #love #art #style #travel #followme #fitness #beautiful #smile #music #girl #vapes #vapestagram... Exclusive product for adults. This product contains nicotine, addictive substance. Do not share content with minors.
Health claims	N/A	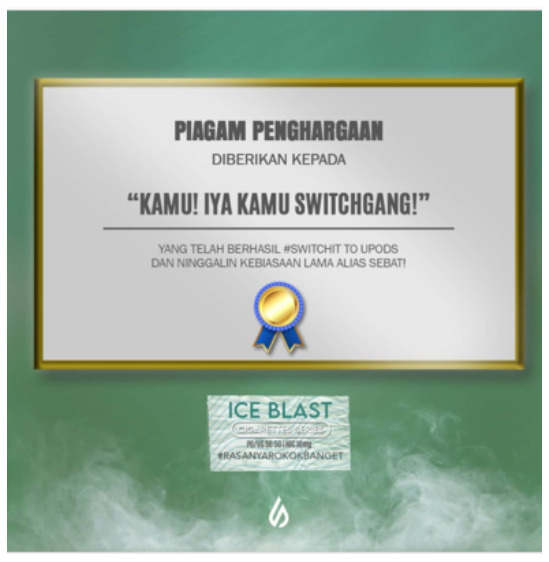 Source: upods_id Instagram page https://www.instagram.com/p/CYTQE-jvhCY/	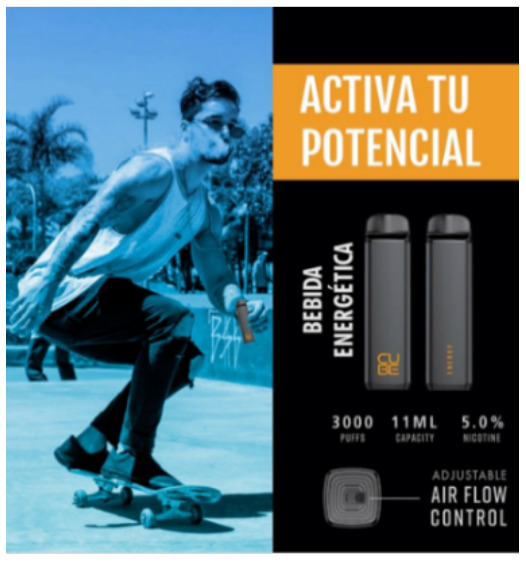 Source: kapitalsmokeandvapor Instagram page https://www.instagram.com/p/CY2bzzvDogK/
Caption translation		Happy yes for you who have managed to stop smoking! For those of you who are still struggling, hopefully it can stop the habit as soon as possible. Don't forget, upods are always present as an alternative cigarette for those of you who want to stop smoking!	What effects does the vape have in the physical skills of athletes? 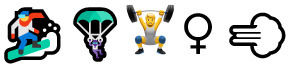 When you think about professional athletes, you do not come to mind smoking or even vape, but there are hundreds of athletes who use vapes. Those who smoked before vape, quickly realized that they had a much better pulmonary capacity and their resistance went out in a matter of weeks. In addition, former smokers experienced much more strength after quitting smoking and noted that they can raise weights significantly larger than before.... exclusive product for graders. This product contains nicotine, addictive substance. Do not share content with minors.

### Ethics review

This study does not meet the criteria for human subjects review. Our methodology involved the analysis of publicly available marketing content from commercial accounts. There was no involvement of human research participants in the conduct of this research.

## Results

### Prevalence of e-cigarette marketing on social media

A total of 6,337 instances of tobacco marketing were observed during the three-month study period. The volume of tobacco marketing observed in this study was highest in Indonesia, followed by India and Mexico (see Supplementary Figure 1). That said, the largest proportion of e-cigarette marketing relative to other tobacco products was found in Mexico, where it made up 75% of the total volume of tobacco marketing; e-cigarette marketing made up a smaller share of marketing in Indonesia (28%) and India (4%).

In India and Indonesia, marketing for smoking products comprised the largest share of marketing (64 and 69%). In India, with the exception of marketing for bidis, this marketing was surrogate marketing or brand extensions used to indirectly promote smoking products, while in Indonesia, consumer interest groups indirectly promoted smoking products. In Mexico, 22% of marketing was for smoking products (exclusively cigars), which were promoted as indigenous products as part of a proud cultural heritage.

We observed a small proportion of marketing of newer tobacco and nicotine products: Heated tobacco products were marketed in Mexico (2%) and Indonesia (1%) and there were no such instances in India. Marketing for nicotine pouches were observed only in Indonesia (2%).

### Profile or types of accounts promoting e-cigarettes on social media

All the e-cigarette social media content identified for this study was obtained from 35 accounts, including 7 in India, 15 in Indonesia and 13 in Mexico (Appendix Table 2). In Indonesia, the sources of e-cigarette marketing were predominantly product brand accounts (86%), followed by accounts operated by community groups affiliated with product brands (12%) and third-party retailers (2%) (Supplementary Figure 2). In India and Mexico, all accounts (100%) promoting e-cigarettes belonged exclusively to third-party retailers.

In Indonesia, the accounts (Facebook and Instagram) run by community groups were associated with the product brand HexOhm and included a link to more than 100 local chapters across the country. Posts featured group gatherings with members touting HexOhm products and branded clothing and banners (Supplementary Images 1, 2).

In all three countries, accounts were found to frequently promote sales avenues for the purchase of e-cigarettes (see Supplementary Figures 3A-C for examples). In India, Instagram accounts predominantly directed traffic to phone numbers, in particular on WhatsApp, for sales. They tended to claim free and fast delivery nationwide, wholesale options and authentic products. In Mexico, accounts tended to direct traffic to both online stores as well as physical, brick-and-mortar stores. In Indonesia, accounts often included links to their online stores and information on where to pick up products in person; some included the Linktree app which links to the e-commerce sites Tokopedia, Shopee and others. In both Indonesia and Mexico, information on where to buy products in person was often directly provided, whereas it was not in India.

### Marketing tactics used to promote e-cigarettes

In all three countries, direct advertising was the tactic most frequently observed for e-cigarettes (99% in India, 93% in Mexico, 69% in Indonesia; see Supplementary Figure 4). Direct advertising posts explicitly promoted the purchase and use of e-cigarettes, and showed clear images of the product, product brand name and trademark logos (see images in Table 2 for examples).

Price promotions were observed but constituted a small fraction of the overall share of tactics employed in all three countries, ranging from 1% in India to 3% in Indonesia. General marketing to raise the profile of the product brand or company was also observed to a small degree in Indonesia (2%) and Mexico (4%). In Indonesia alone, marketing using events, special occasions and sponsorships constituted a significant share of the tactics used (27%). For example, HexOhm posted images of its community groups during member meetings or at events. Likewise, holiday greetings, such as VOOPOO wishing its followers a happy Nyepi (day of silence) holiday, were posted on behalf of the brand (Table 2). One example of highlighting brand sponsorships through contests includes GeekVape soliciting viewers to predict the Champions League result, in which the football team they sponsor, Paris Saint-Germain, was participating, to win prizes.

### E-cigarette and e-liquid product brands promoted most frequently

We observed a total of 63 product brands, with the greatest variety of e-cigarettes, especially e-liquids, being promoted in Mexico (*N* = 48), and the fewest in India (*N* = 9). Of these, we were able to identify the parent company for 23 different product brands; these product brands and the place of origin of their parent company, is presented in Supplementary Figures 5A,B. Four product brands were marketed in all countries: Vaporesso, VOOPOO, UWELL and SMOK. The country of origin of the products promoted was predominantly China (77%), followed by the U.S. (18%). In Indonesia alone, we found one instance of a locally owned e-cigarette brand being promoted (4%).

### Social media channels used for e-cigarette marketing and engagement by platform

In all three countries, most marketing occurred on Meta platforms, particularly Facebook and Instagram (Supplementary Figure 6A). In India, all e-cigarette marketing was observed on Instagram (100%). In Mexico, e-cigarette marketing was mostly observed on Facebook (56%), followed by Instagram (43%). In Indonesia, e-cigarette marketing was mostly observed on Facebook (51%), followed closely by Instagram (48%).

We also measured the platforms on which the marketing received the highest engagement, which captures the sum of likes/loves, reshares and replies/comments (Supplementary Figure 6B). In India, all posts were observed on Instagram, and therefore we could not measure if platforms generated different engagement rates. In Indonesia, posts on YouTube (1%) generated the highest average engagement per post (484), whereas in Mexico, posts observed on TikTok (1%) had the highest average engagement (89).

The videos on YouTube in Indonesia that generated high engagement were created by “e-cigarette reviewers” who tried out different products on behalf of retailers. They used an episode format, where the hosts reviewed e-cigarette devices and tried e-liquid flavors, while trading jokes. On TikTok in Mexico, most videos featured the unboxing of e-cigarette devices with popular songs playing in the background; in one of the videos, the viewer was told that this was their sign to stop smoking cigarettes for good and suggested that the company could recommend “e-cigarette” kits.

We also analyzed how the type of social media account related to the choice of platform used (Supplementary Figure 6C). This was most relevant to Indonesia where there were a variety of different types of accounts. We observed that third-party retailers tended to promote e-cigarettes on Instagram (56%) and YouTube (44%), while product brand accounts mostly used Facebook (52%) and brand-associated community groups used Instagram (52%).

### Message framing used for e-cigarette marketing and engagement by message type

In all three countries, “product features” was the frame by which e-cigarettes were predominantly marketed: 86% of posts in India, 58% in Indonesia and 73% in Mexico used this frame (Supplementary Figure 7A). These posts tended to highlight features such as: device colors and a “stylish,” “modern,” “luxurious” and “futuristic” design; technical specifications such as design that prevents air flow leakage and the number of puffs per unit for disposable devices; and the convenience and usability of the products, such as touch screen indicators of battery life, milliamp-hours or voltage, and the portability of devices for daily use. The customizability of products was emphasized and e-liquid flavors were highlighted to appeal to different tastes, including playful takes on minty or fruit flavors such as “mean mango,” “fruit punch,” “peach pleasure” and “blue slushee (Supplementary Image 3).” Other e-liquid names include “cheezz delight,” “cola man” and “custard monster,” which refer to ultra-processed foods and drinks.

The next most commonly used message frame varied across countries: in India, it was entertainment (13%); in Indonesia, it was informational, which included instruction on how to use products or provided information on the product brand or company (14%); and, in Mexico, it was health claims (8%). In Mexico, the hashtag #elvapeosalvavidas (#vapingsaveslives) was used in 48% of posts that promoted the health benefits of e-cigarettes over combustible cigarettes. Posts that promoted e-cigarettes as being healthier than combustible cigarettes were very uncommon in Indonesia (0.3%) and non-existent in India. See Table 3 for examples from each country.

We measured the type of message framing that had the highest average audience engagement, which is calculated by combining likes/loves, shares and comments/replies (Supplementary Figure 7B). Average engagement may serve as a proxy of the effectiveness of online marketing strategies, however, this measure is sensitive to outliers and needs to be interpreted carefully. In India, one post providing information on Juul products received the highest average engagement (2,933), however, given that this one post skewed the distribution of engagement metrics, we determined that overall, on average, posts touting product features generated the highest average engagement in India (132). In Indonesia, on average, entertaining posts generated the most engagement (322); this largely included reposted videos of users doing tricks with e-cigarettes, as well as humorous videos. In Mexico, posts making health claims about e-cigarettes being safer than combustible cigarettes had the highest average engagement (14).

## Discussion

Our study found several common features in e-cigarette marketing in India, Indonesia and Mexico, but also some important distinctions that likely reflect the political, economic and social contexts of the countries.

E-cigarette marketing was prevalent in all three countries but it varied in volume and share of overall tobacco marketing. The volume of e-cigarette marketing was highest in Indonesia, followed by Mexico and India. However, as a share or percentage of total tobacco marketing, e-cigarette marketing was highest in Mexico, where it dwarfed marketing of other tobacco products (75%). In India and Indonesia, which have among the world's largest smoking tobacco markets that include indigenous products like bidis and kreteks (54, 55), e-cigarette marketing made up a smaller share of the overall marketing (28% in Indonesia and 4% in India).

The regulatory environments in the three countries may explain the patterns of marketing observed. At the time of this study, regulations were strongest in India, where e-cigarettes were comprehensively banned (42). They were weakest in Indonesia, which has neither strong tobacco control regulations nor any governing e-cigarettes (46). And were in flux in Mexico, where regulations on tobacco products and e-cigarettes were strengthened both during and after the study (44). The differences in regulatory environment appear to be mirrored in the observed patterns of marketing in the three countries. E-cigarette marketing was lowest in India, whereas, consistent with the lax regulatory environment in Indonesia, not only did we observe a high volume of e-cigarette marketing there but also a varied mix of products, like heated tobacco products and nicotine pouches. In fact, the marketing observations in our study are consistent with the openness of the Indonesian market to newer products where British American Tobacco/Bentoel's Velo, Indonesia's first nicotine pouch, and Philip Morris International/Sampoerna's heated tobacco product, IQOS, have launched (56, 57). In Mexico, consistent with its evolving regulatory environment at the time of this study, we observed a volume of marketing that fell between that in India and Indonesia. The overall level of regulation and marketing observed also matched up with e-cigarette use rates in each country, which were highest in Indonesia (3%) and lowest in India (0.02%) (37, 39).

The regulatory environment may also have had an effect on the profile of marketers in the three countries. These were exclusively third-party retailers in India and Mexico—we found no instances of product or company brands promoting e-cigarettes in these two countries. In India, where e-cigarettes are strictly banned, viewers were encouraged to reach out to retailers more covertly using provided telephone numbers, often via WhatsApp. We did not observe accounts linking to websites, despite evidence that shows that online e-cigarette retailers were still prevalent after the e-cigarette ban (58). In Mexico, accounts often provided links to online stores, along with other contact methods, like emails and telephone numbers, and locations of physical stores. In contrast, in Indonesia, e-cigarettes were primarily promoted by product brand accounts, followed by brand-affiliated community groups and third-party retailers. Indonesian accounts frequently provided links to online stores and other contact methods, as well as to Linktree pages that connect viewers to a range of e-commerce apps where purchases can be made.

Formal tobacco enterprises, particularly transnational companies with significant business interests, are less prone to be seen to take the risk of violating regulations—or incurring regulations in countries where this is a possibility—by explicitly promoting e-cigarettes. It is possible that in such countries as India or Mexico, global tobacco companies may be using more covert means of promotion, such as through the use of influencers, or ostensibly by backing the third-party retailers that promote these products, a tactic seen in the promotion of cigarettes (59). Third-party retailers, on the other hand, are likely to be fragmented and more easily able to avert oversight. This was evident in how their promotions clearly pictured products with brand logos via public accounts, suggesting a lack of concern with discovery. Thus, our data suggests the importance of monitoring efforts that investigate covert means of promotion in countries with existent or forthcoming regulations. It also suggests the importance of considering the role played by third-party retailers in the promotion of e-cigarettes and the utility of complementary interventions, such as vendor licensing, to stop their proliferation.

Direct advertising to promote purchase was the predominant purpose of e-cigarette marketing in all three countries. We found that product features, known from previous studies to appeal to youthful tastes, were highlighted (60, 61). The posts appealed to youth desire for a variety of e-liquid flavors. They emphasized the look of the devices, such as the variety of colors in which they were available. And they highlighted the technological advances of the products, such as battery life, appealing to the youthful orientation toward new technology (62, 63).

E-cigarettes were also associated with favorable cultural representations in the posts observed in our study, thus attempting to normalize their use. Sales promotions in both Indonesia and Mexico were frequently tied to holidays, and a culture of giving e-cigarettes as gifts was promoted. E-cigarettes were glamorized in posts in Indonesia to promote their desirability. This form of “social selling” is often geared toward youth and new users (64) and attempts to establish new social norms (65).

Harm reduction messaging was less prevalent than expected in all three countries. Direct harm reduction messaging was most common in Mexico (8%), followed by Indonesia (0.3%), and was not observed in India at all (0%). The prominence of explicit harm reduction messaging in Mexico, frequently accompanied by the hashtag #elvapeosalvavidas (vaping saves lives) may have been an attempt to rally e-cigarette users to counter government efforts during this period to restrict e-cigarettes. In fact, in May 2022, in the months following the period of analysis reported in this paper, Mexico enacted a complete ban on e-cigarettes (66).

The message framing that garnered the most engagement also varied by country: In India, where directly selling products appeared to be the primary goal of most posts, product features garnered the most engagement, whereas in Indonesia, where accounts engaged more in social selling, entertaining posts had the highest engagement. This included many user-submitted videos of e-cigarette tricks, which have been associated with e-cigarette use (67). In Mexico, amid the national policy debate on e-cigarettes, posts that positioned e-cigarettes as a smoking harm reduction tool received the most engagement.

The message frames used for the promotion of e-cigarettes observed in our study have several important implications for tobacco control efforts. Foremost, they underscore attempts to normalize e-cigarette use in these countries, suggesting the need for strong counter-marketing efforts by governments and tobacco control campaigners. Furthermore, they call into question the argumentation frequently made by e-cigarette promoters for e-cigarettes as a harm reduction device. Our study presents evidence of e-cigarettes being promoted most often as a desirable product and habit, and not as a harm reduction aid, which is the argument frequently used to claim regulatory exemptions for e-cigarettes (68). Governments and public health practitioners would be well-advised to question the premise for e-cigarettes given the inconsistency in its promotion.

Meta platforms, including Facebook and Instagram, were the most frequently used platforms for promotion. The opacity of WhatsApp provides a cover for the covert sales of e-cigarettes, despite bans being in place in countries like India. Our study highlights the relative ineffectiveness of company policies, like those by Meta, in reducing violations. Improved monitoring and reporting of tobacco marketing in these platforms is warranted.

Finally, our study highlights the generally diffused nature of e-cigarette product brands. We found 63 e-cigarette and e-liquid product brands marketed across the three countries, but only four e-cigarette product brands that were marketed in all three countries: Vaporesso, UWELL, VOOPOO and SMOK. We did not identify any e-cigarette product brands owned by global tobacco companies or their subsidiaries. In our study, of the products that we could identify parent companies for, 77% of the e-cigarette product brands being marketed were of companies with headquarters in China, where 95% of the world's e-cigarettes are manufactured (69); another 18% came from brands owned by U.S.-headquartered companies; and there was one locally-owned company in Indonesia (4%). Our study findings are consistent with what has been observed of the e-cigarette industry as a diversified—there were more than 460 e-cigarette product brands on the global market as of 2017—and opaque industry that makes identifying, tracking and regulating key players both a challenge and a necessity (70).

### Limitations

There are limitations to our study worth noting. First, our study was by design, purposive in its selection of social media accounts and while every effort was made, *via* expert inputs, crowdsourcing, and systematic complementary searchers, to make the lists exhaustive, our findings may not be representative of the full extent and breadth of online e-cigarette marketing within the three countries studied. For instance, our study may not have picked up the highly covert ways of online promotion, such as through the use of influencers, targeted paid ads, peer-to-peer marketing, or through private groups that online search tools, like Radarr, cannot access. TERM collects shared and earned media, which offer potential for wide exposure, but the monitoring system does not collect paid or owned media, which are also important parts of the marketing model (71). The rapid, implementation-focused approach of our study is intended to provide policymakers with dipstick indications of the evolving nature of tobacco marketing present in their contexts. Future studies using comprehensive surveillance methods would be important to more fully explore the scale of e-cigarette marketing present in these countries.

Second, our study focused on identifying and analyzing marketing content explicitly directed at these countries. Given the cross-border nature of social media, it is possible that online users in the three countries in our study were exposed to global campaigns of transnational corporations or online activities occurring in other countries. In a related vein, it cannot be claimed that the list of accounts monitored by our study was entirely complete for those countries. As evident by the wide variety of product brands unearthed by our study, the market for e-cigarettes is evolving and relatively opaque. Multiple studies of varied methodologies, including surveys of online users to measure exposure and studies of retail sales, will be required to triangulate data and create a fuller picture of the extent of e-cigarette marketing that is prevalent online.

Third, this study used data collected during a limited study period of 3 months, and therefore cannot offer insights into marketing practices that occur outside of that period. However, TERM is a continuous media monitoring system that presents data *via* regular reports that can be used to supplement findings in this study (47).

Finally, the current approach to the selection and analysis of content is text-based. This would mean that any content that solely contained images would not be included for analysis. This limitation in the search parameters may have missed accounts or posts on heavily visual media such as YouTube or TikTok, where captions and hashtags are less often provided. This would mean that our study findings may have overrepresented Meta platforms, including Facebook and Instagram, and underrepresented the role of platforms like YouTube and TikTok in promoting e-cigarettes. However, given that Meta platforms, and particularly Instagram, continue to be the platforms of choice for e-cigarette-related content (20) and that little to nothing was known about the type of e-cigarette marketing on these platforms in the three countries studied, our findings add significant value. Future studies might consider how to better incorporate imagery in the identification of instances of online tobacco marketing.

## Conclusion

Our findings have several policy implications. First and foremost, our study highlights the importance of rapid and continuous digital media monitoring to track and respond to the presence of tobacco marketing online. Our study shows a presence of online marketing and a variety of promoters and brands, suggesting an essentially opaque environment requiring much further discovery. Agile systems, like TERM, that are built on crowdsourced and expert inputs, can play a crucial role in complementing the more comprehensive surveillance efforts present in countries to respond to the constantly evolving industry tactics in digital environments. Our findings underscore the importance for tobacco control stakeholders, from governments to researchers and advocates that monitor the industry, to consider how a range of methods can be pooled and applied to triangulate information on tobacco marketing and industry behavior.

Second, our study suggests the inconsistency in arguments made for e-cigarettes. While e-cigarettes are promoted in policy dialogues as a means of harm reduction, it is evident from our study that e-cigarettes are presented in marketing, particularly to youth, as desirable consumables—not as a health aid. This is evident from the appealing flavoring, attractive presentation, and staging of e-cigarettes as technologically cutting edge. Our study underscores the need for governments, media and tobacco control advocates to call into question the public health justifications used by the e-cigarette industry, and the necessity of efforts to counter-message efforts to normalize e-cigarette and tobacco use.

Finally, our study highlights the importance of clear and comprehensive regulation. Our findings show that bans or restrictions on e-cigarettes may help effectively curb online e-cigarette marketing, particularly marketing originating directly from product brands. That said, our findings of third-party retail promoters in countries like India with strong regulations suggests the need for a comprehensive and complementary set of policies to prevent violations that may otherwise slip under the radar.

In conclusion, our study provides important insights for tobacco control stakeholders from governments, researchers, advocates to the media, on the evolving nature of e-cigarette marketing in low and middle-income countries and suggests the importance of continuous monitoring to keep up with industry practices and to strengthen counter-response.

## Data availability statement

The raw data supporting the conclusions of this article will be made available by the authors, without undue reservation.

## Author contributions

NM: conception, study design and direction of data analysis, data interpretation, and writing. MM: study design and direction of data analysis, data interpretation, literature review, and writing. HR: study design, data interpretation, literature review, and writing. SK, SD, and CM-M: data analysis. R and BA: review of writing. All authors contributed to the article and approved the submitted version.

## Funding

The TERM study is a project of Vital Strategies' Tobacco Control Program, which receives funding from Bloomberg Philanthropies.

## Conflict of interest

Authors NM, MM, HR, SK, SD, CM-M, R, and BA were employed by Vital Strategies.

## Publisher's note

All claims expressed in this article are solely those of the authors and do not necessarily represent those of their affiliated organizations, or those of the publisher, the editors and the reviewers. Any product that may be evaluated in this article, or claim that may be made by its manufacturer, is not guaranteed or endorsed by the publisher.

## Supplementary material

Supplementary Image 1 A HexOhm Mojokerto community meet in Indonesia promoted *via* the product brands' Instagram page.

Supplementary Image 2 A HexOhm Palu community meet in Indonesia promoted *via* the product brands' Instagram page.

Supplementary Image 3 Examples of different types of flavors used to market e-cigarettes.

Supplementary Figure 1 Volume and proportion of tobacco marketing by product type in India, Indonesia, and Mexico.

Supplementary Figure 2 Percentage of e-cigarette marketing by account type in India, Indonesia, and Mexico.

Supplementary Figure 3 **(A–C)** Avenues for purchase of e-cigarettes promoted in Instagram posts in India, Indonesia, and Mexico.

Supplementary Figure 4 Marketing tactics used to promote e-cigarettes in India, Indonesia and Mexico.

Supplementary Figure 5 **(A)** E-cigarette product brands with known parent companies marketed in India, Indonesia, and Mexico. **(B)** Origin of parent companies of e-cigarette product brands with known parent companies marketed in India, Indonesia, and Mexico.

Supplementary Figure 6 **(A,C)** Percentage of e-cigarette marketing by social media platforms in India, Indonesia, and Mexico. **(B)** Total engagement and average engagement by platform in India, Indonesia, and Mexico.

Supplementary Figure 7 **(A)** Volume of e-cigarette marketing by message framing in India, Indonesia, and Mexico. **(B)** Total engagement and average engagement by message framing in India, Indonesia, and Mexico.
