# Supplementary figures and images for: A content analysis of e-cigarette marketing on social media: Findings from the Tobacco Enforcement and Reporting Movement (TERM) in India, Indonesia and Mexico
Source: Front Public Health. 2022 Nov 8;10:1012727. doi: 10.3389/fpubh.2022.1012727 (PMC9679495; doi:10.3389/fpubh.2022.1012727)

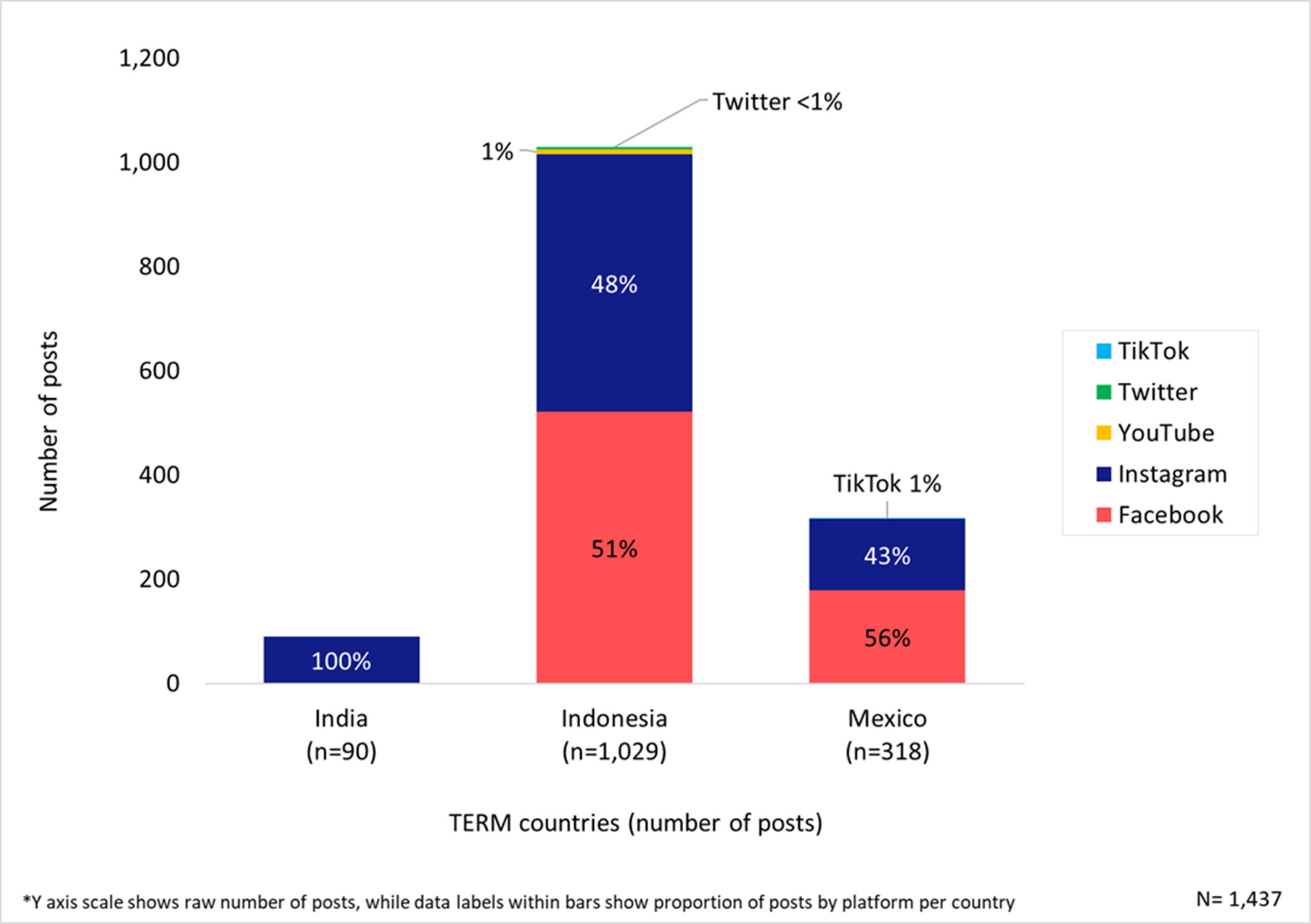

Supplement: Supplementary Image 1 — A HexOhm Mojokerto community meet in Indonesia promoted via the product brands' Instagram page. [file Data_Sheet_1.ZIP › Supplementary Material Presentation/Figure6a_Percentage of e-cigarette marketing by social media platforms in India, Indonesia and Mexico.tif]

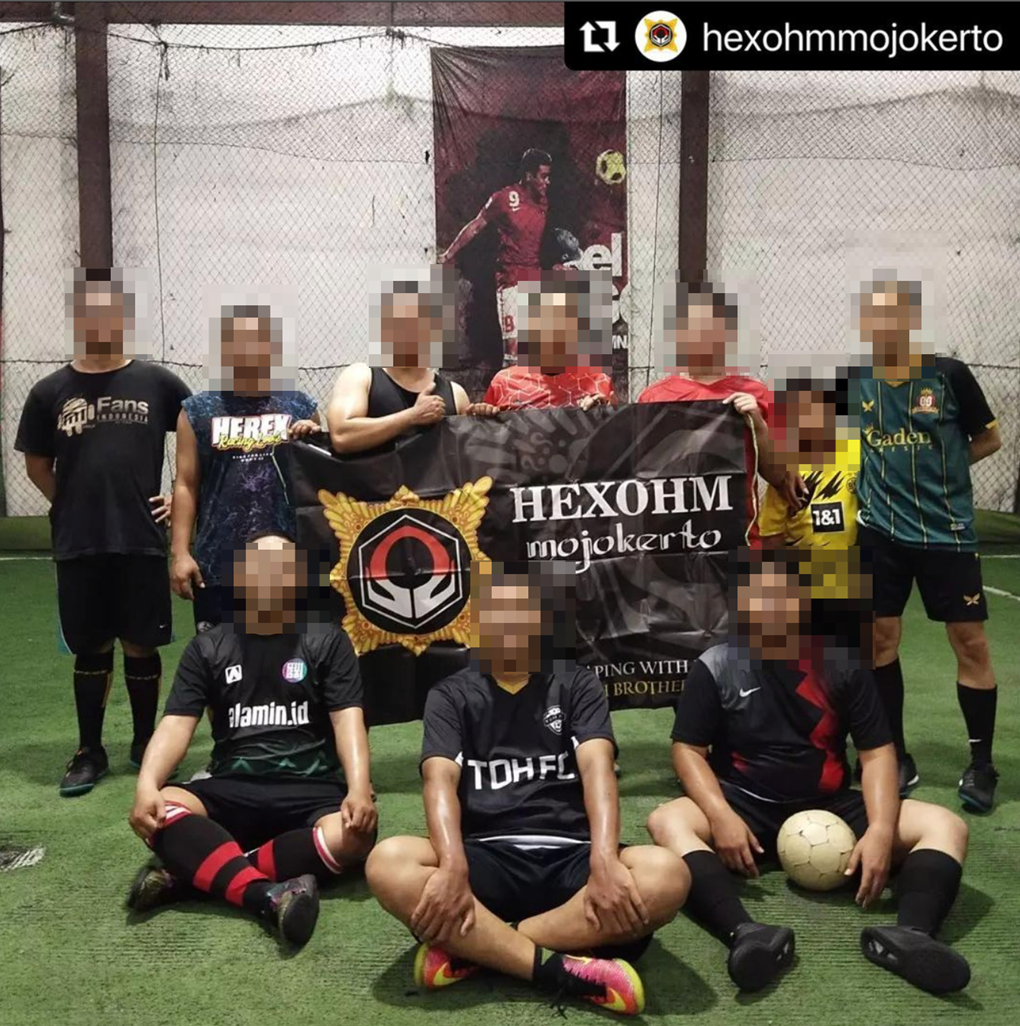

Supplement: Supplementary Image 1 — A HexOhm Mojokerto community meet in Indonesia promoted via the product brands' Instagram page. [file Data_Sheet_1.ZIP › Supplementary Material Presentation/Image 1_A HexOhm Mojokerto community meet in Indonesia promoted via the product brandsΓÇÖ Instagram page.tif]

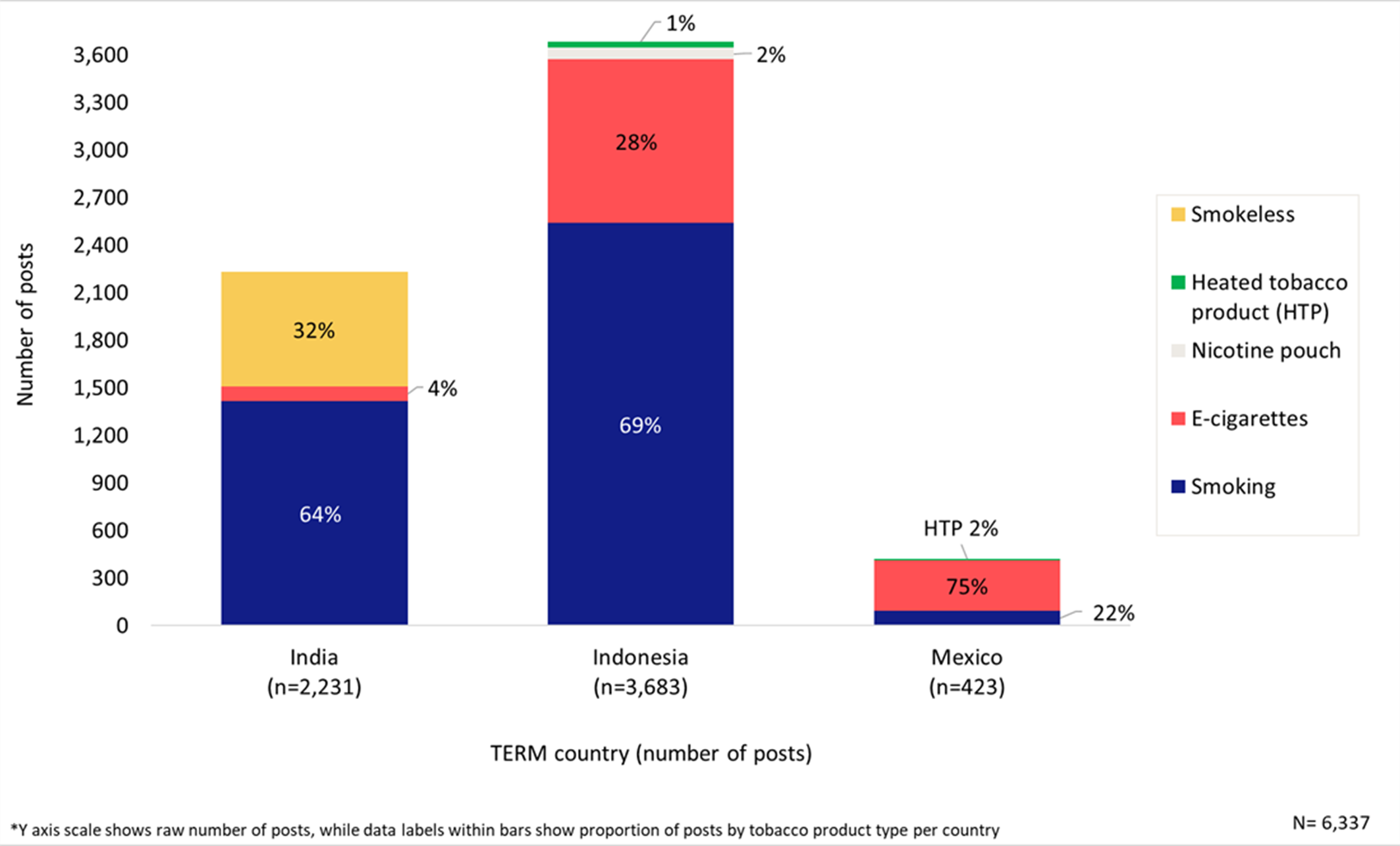

Supplement: Supplementary Image 1 — A HexOhm Mojokerto community meet in Indonesia promoted via the product brands' Instagram page. [file Data_Sheet_1.ZIP › Supplementary Material Presentation/Figure1_Volume and proportion of tobacco marketing by product type in India, Indonesia and Mexico.tif]

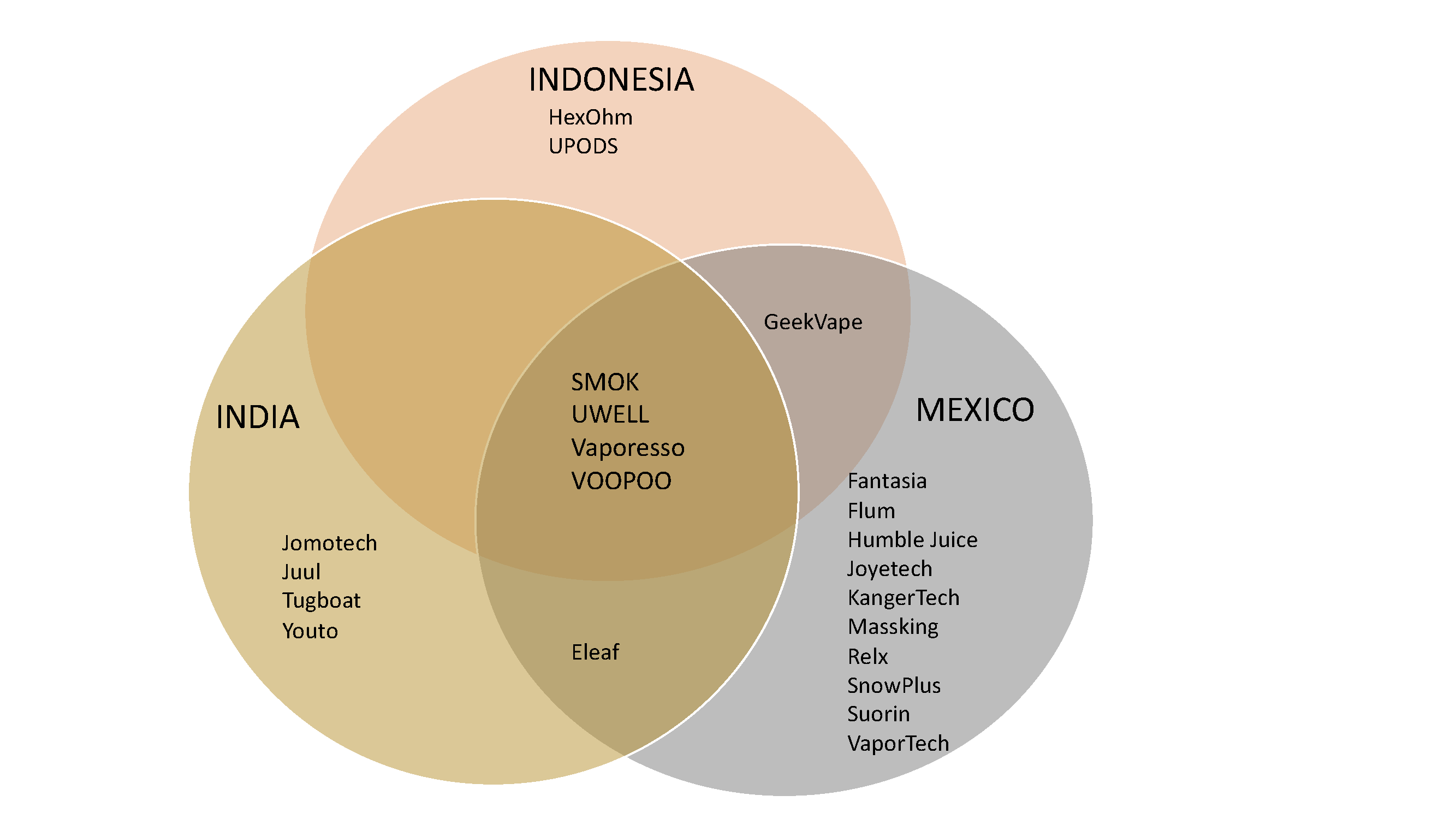

Supplement: Supplementary Image 1 — A HexOhm Mojokerto community meet in Indonesia promoted via the product brands' Instagram page. [file Data_Sheet_1.ZIP › Supplementary Material Presentation/Figure 5a_Top marketed e-cigarette product brands in India, Indonesia and Mexico_Page_2.tiff]

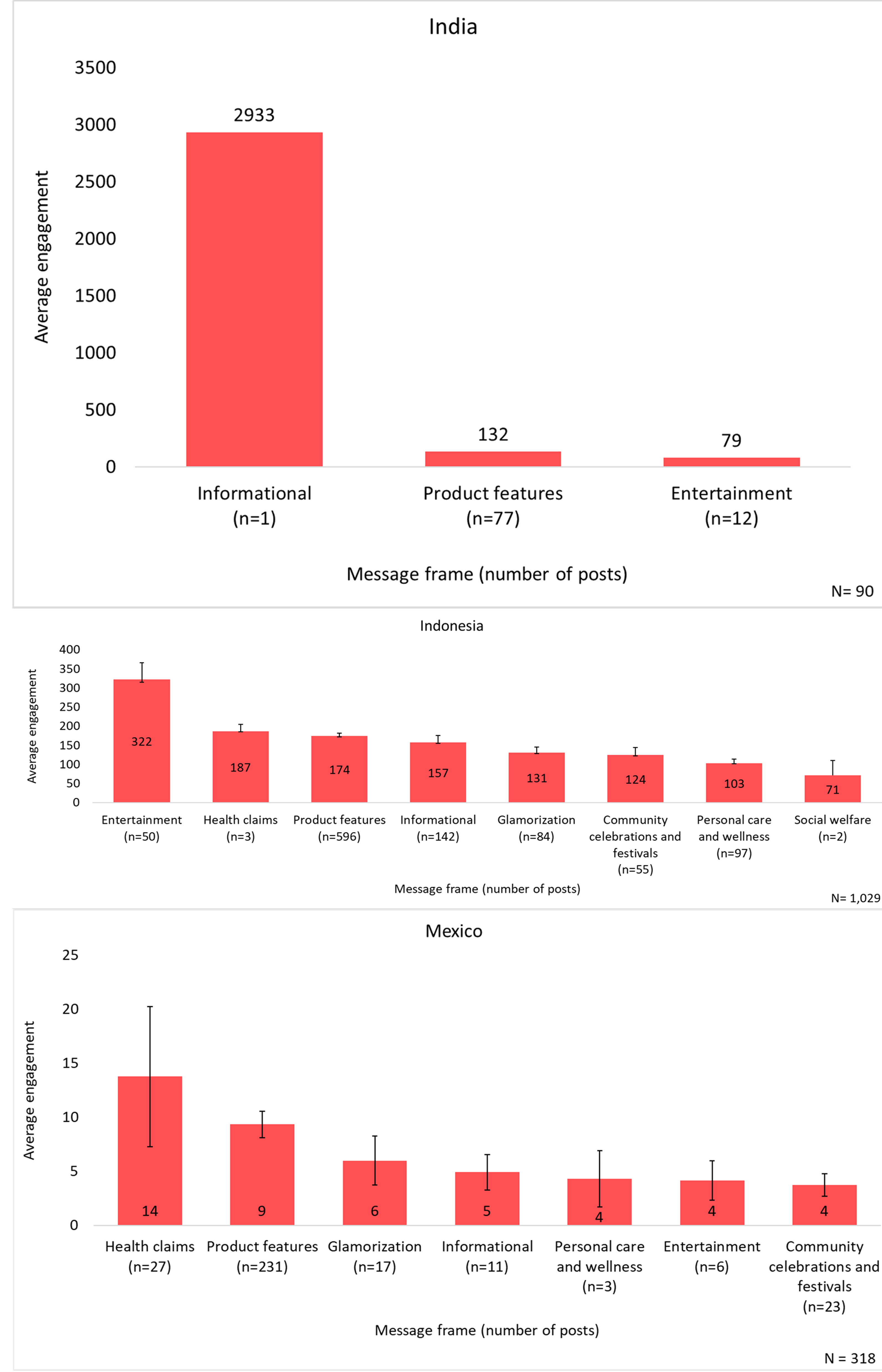

Supplement: Supplementary Image 1 — A HexOhm Mojokerto community meet in Indonesia promoted via the product brands' Instagram page. [file Data_Sheet_1.ZIP › Supplementary Material Presentation/ Figure7b_Total engagement and average engagement by message framing in each country.tif]

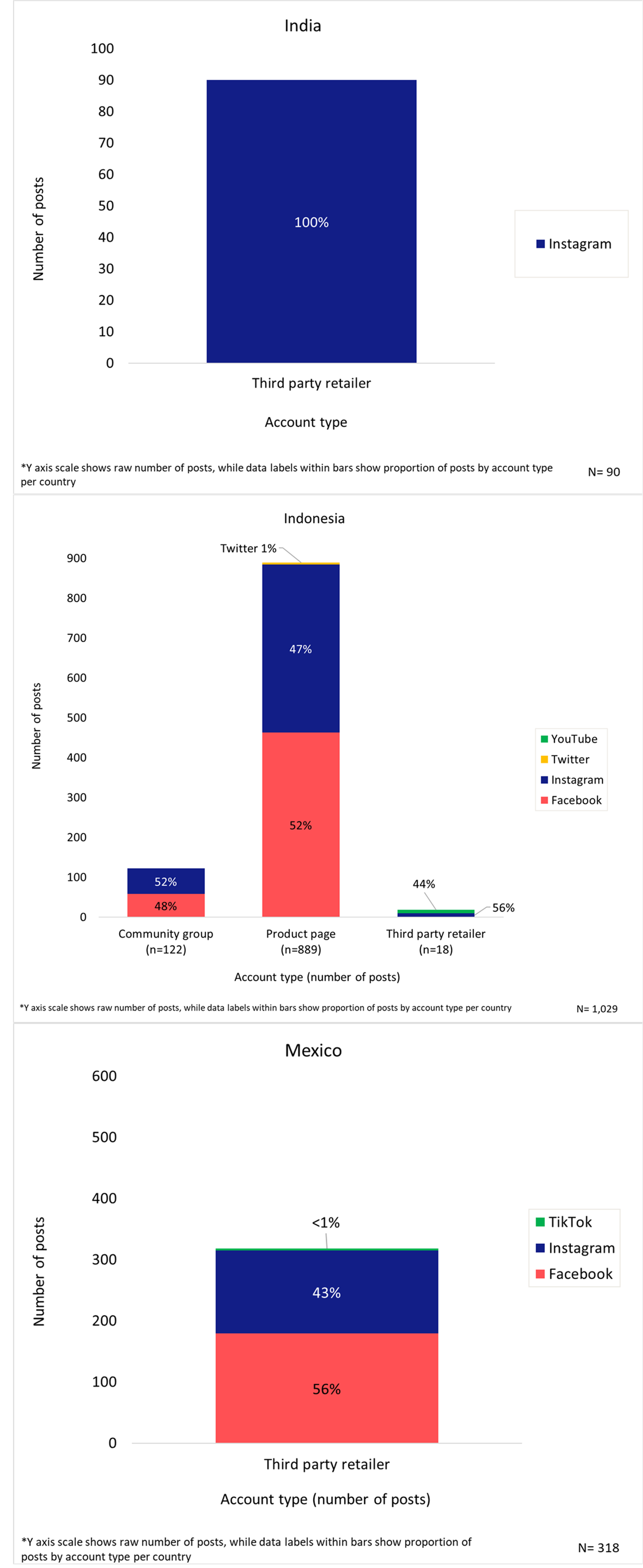

Supplement: Supplementary Image 1 — A HexOhm Mojokerto community meet in Indonesia promoted via the product brands' Instagram page. [file Data_Sheet_1.ZIP › Supplementary Material Presentation/Figure6c_Percentage of e-cigarette marketing by social media platforms and account type in India, Indonesia, and Mexico.tif]

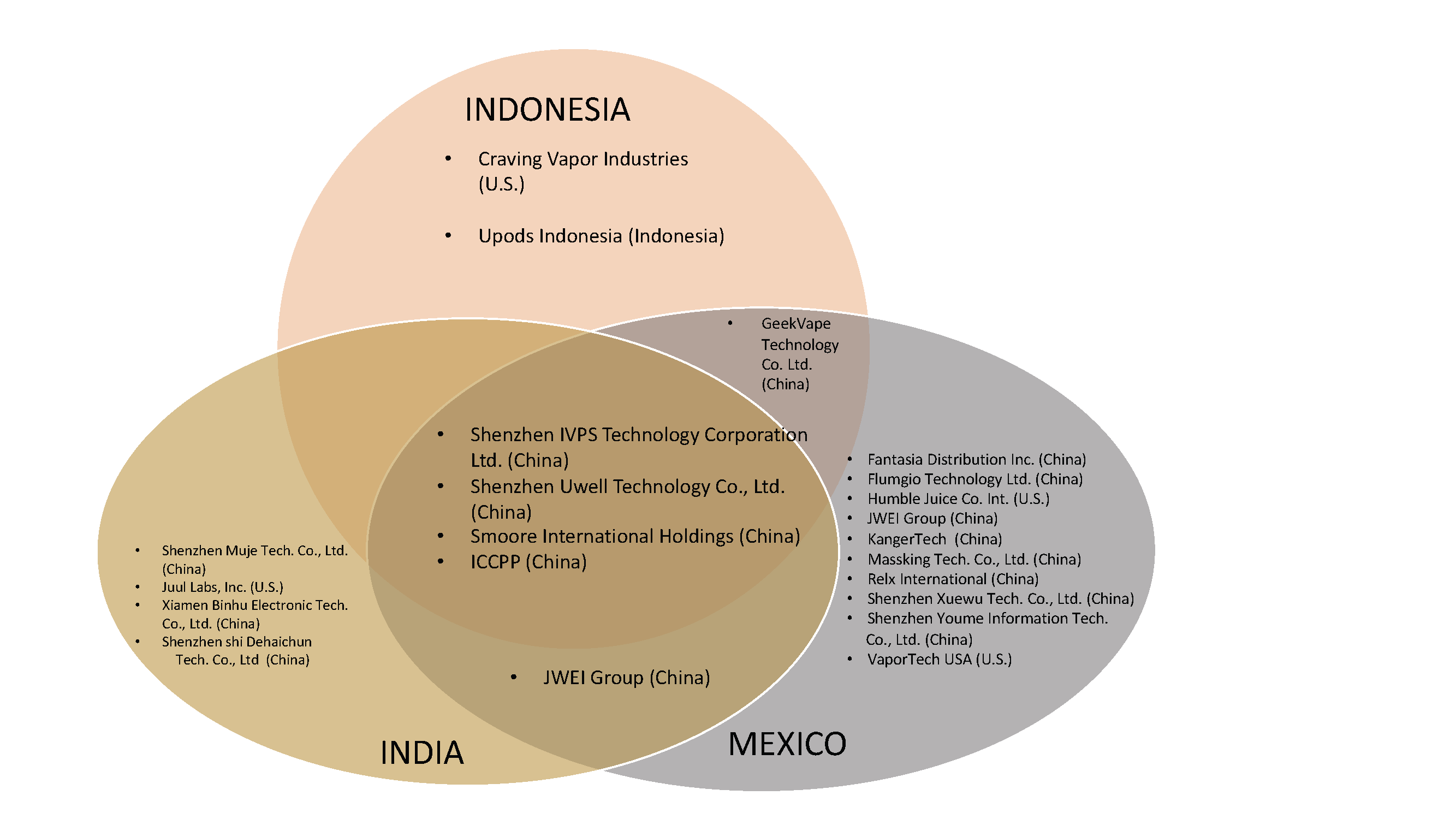

Supplement: Supplementary Image 1 — A HexOhm Mojokerto community meet in Indonesia promoted via the product brands' Instagram page. [file Data_Sheet_1.ZIP › Supplementary Material Presentation/Figure 5b- Origin of parent companies of top marketed e-cigarette product brands in India, Indonesia and Mexico_Page_1.tiff]

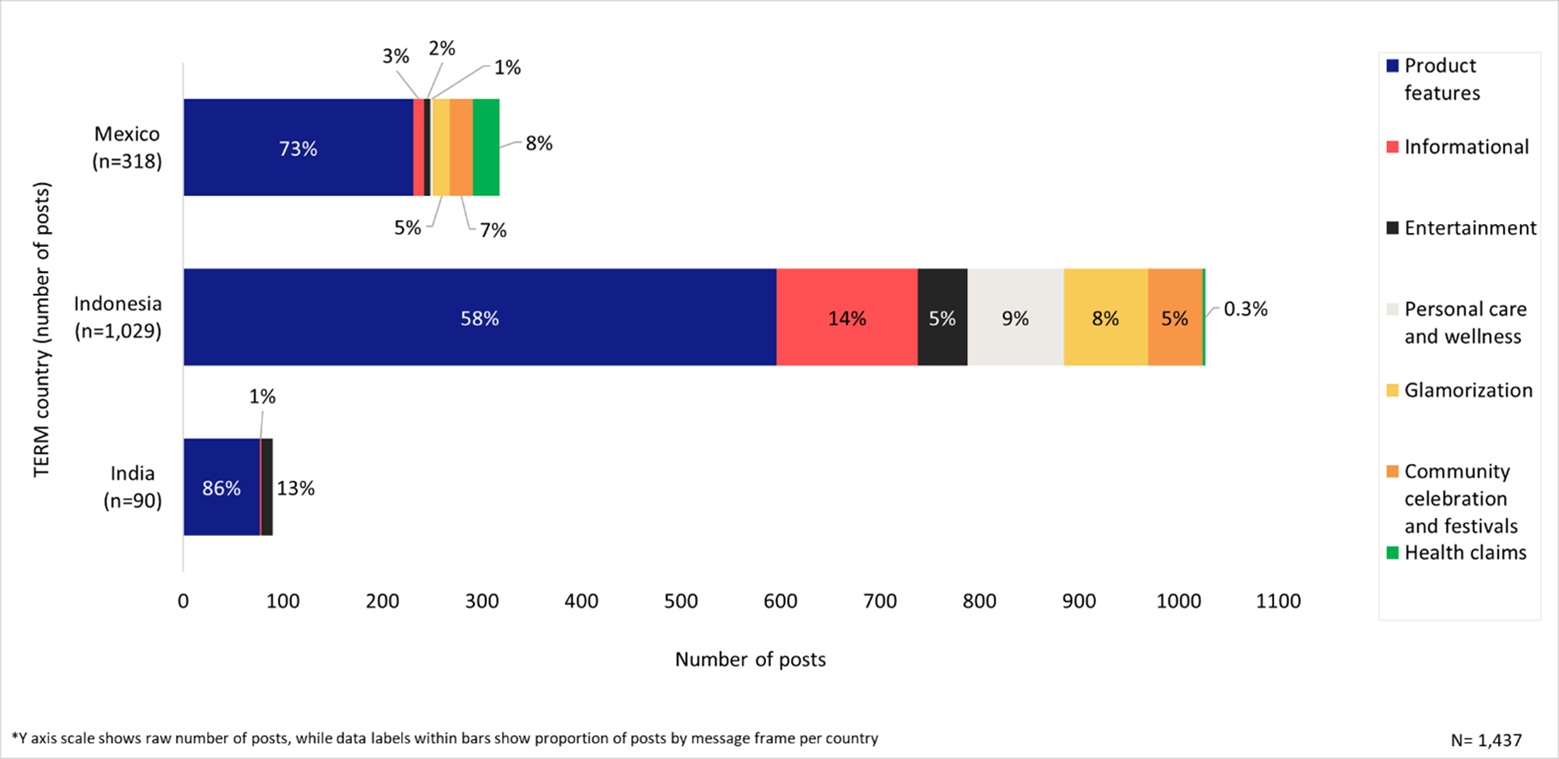

Supplement: Supplementary Image 1 — A HexOhm Mojokerto community meet in Indonesia promoted via the product brands' Instagram page. [file Data_Sheet_1.ZIP › Supplementary Material Presentation/Figure7a_Volume of e-cigarette marketing by message framing in India, Indonesia and Mexico.tif]

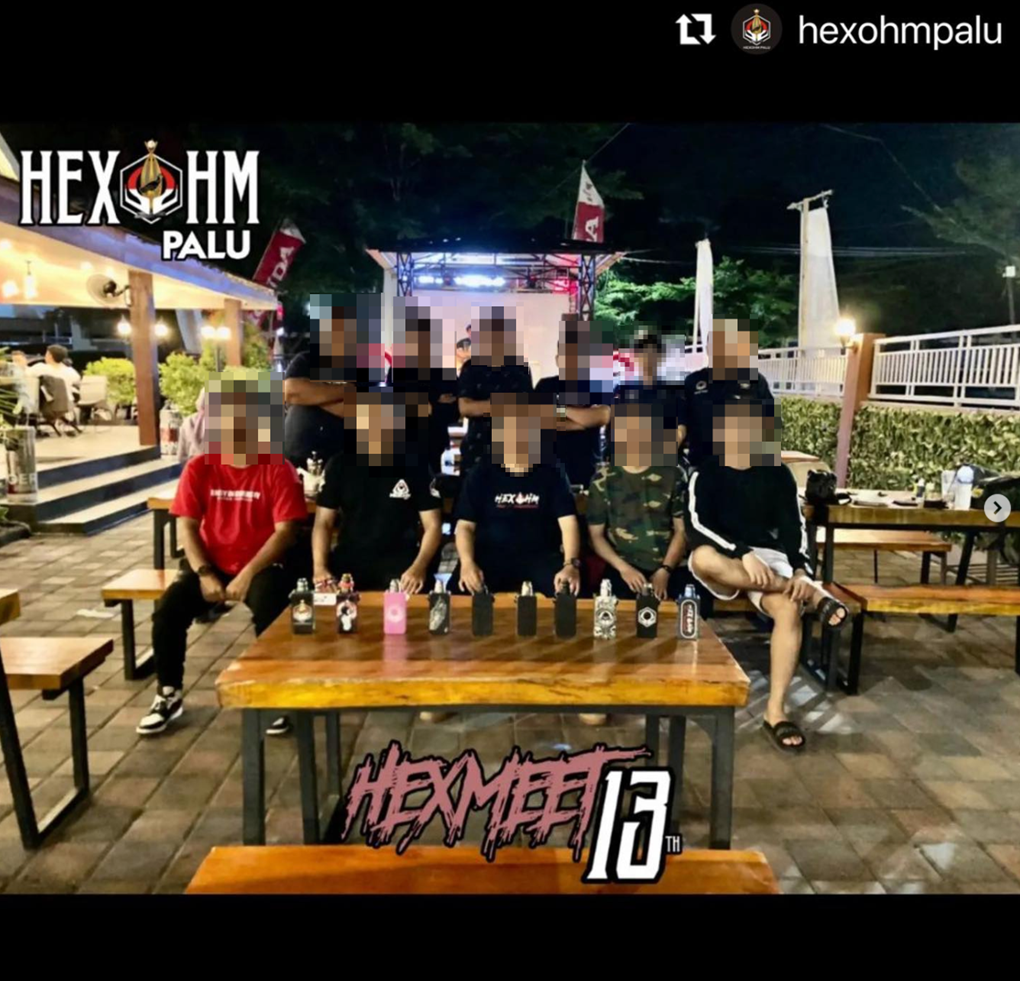

Supplement: Supplementary Image 1 — A HexOhm Mojokerto community meet in Indonesia promoted via the product brands' Instagram page. [file Data_Sheet_1.ZIP › Supplementary Material Presentation/Image 2_A HexOhm Palu community meet in Indonesia promoted via the product brandsΓÇÖ Instagram page.tif]

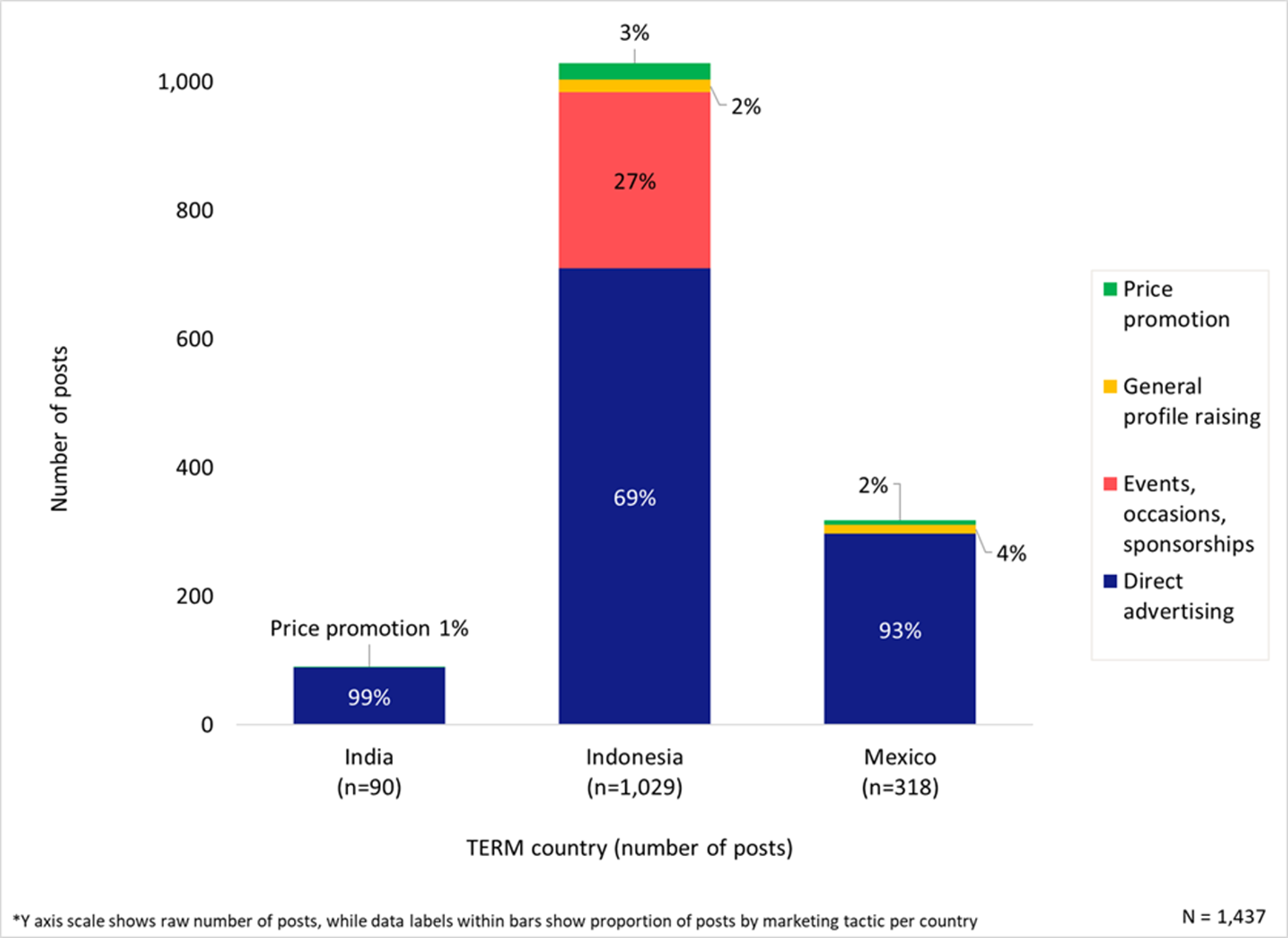

Supplement: Supplementary Image 1 — A HexOhm Mojokerto community meet in Indonesia promoted via the product brands' Instagram page. [file Data_Sheet_1.ZIP › Supplementary Material Presentation/Figure 4_Marketing tactics used to promote e-cigarettes in India, Indonesia and Mexico.tif]

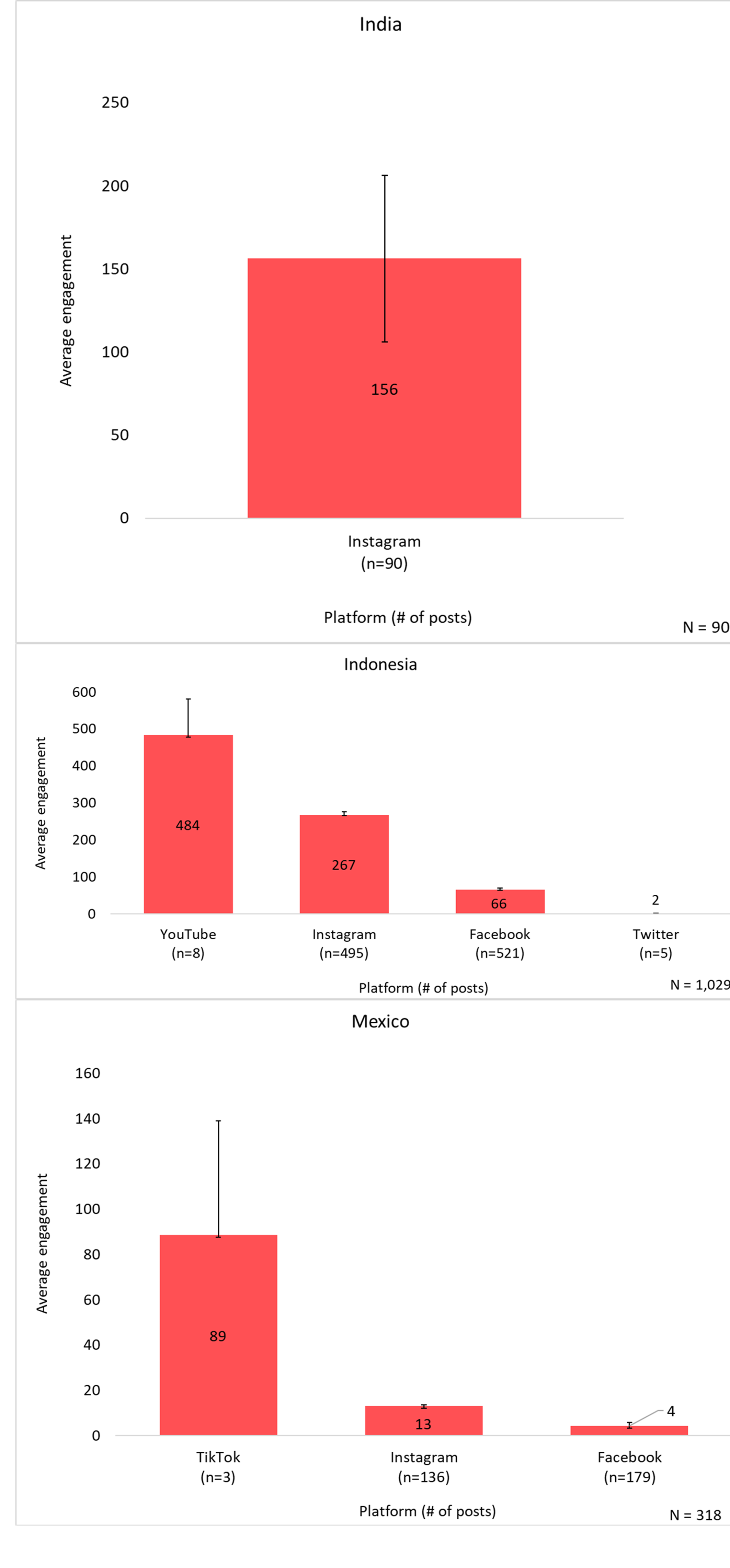

Supplement: Supplementary Image 1 — A HexOhm Mojokerto community meet in Indonesia promoted via the product brands' Instagram page. [file Data_Sheet_1.ZIP › Supplementary Material Presentation/Figure6b_Total engagement and average engagement by platform in India, Indonesia and Mexico.tif]

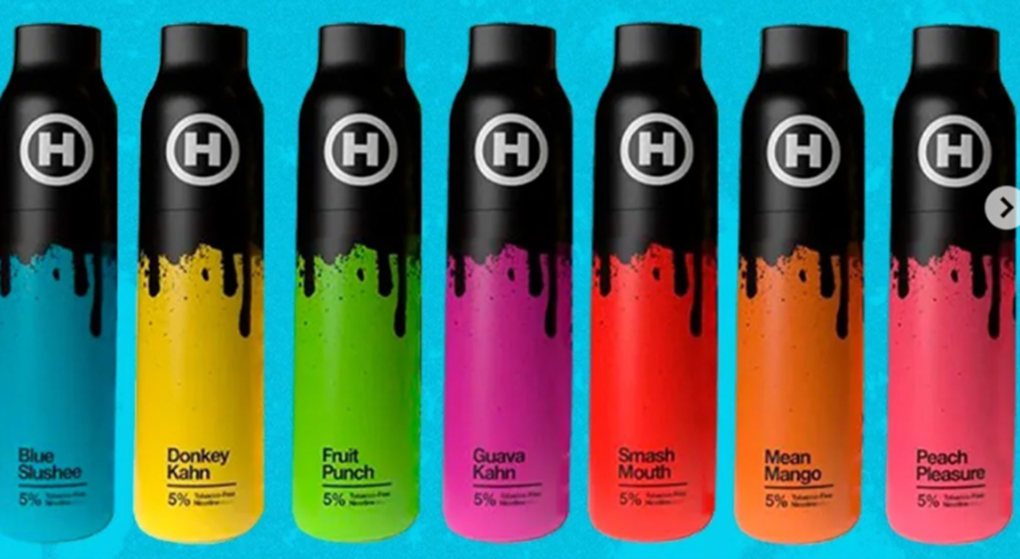

Supplement: Supplementary Image 1 — A HexOhm Mojokerto community meet in Indonesia promoted via the product brands' Instagram page. [file Data_Sheet_1.ZIP › Supplementary Material Presentation/Image 3_Examples of different types of flavors used to market e-cigarettes.tif]

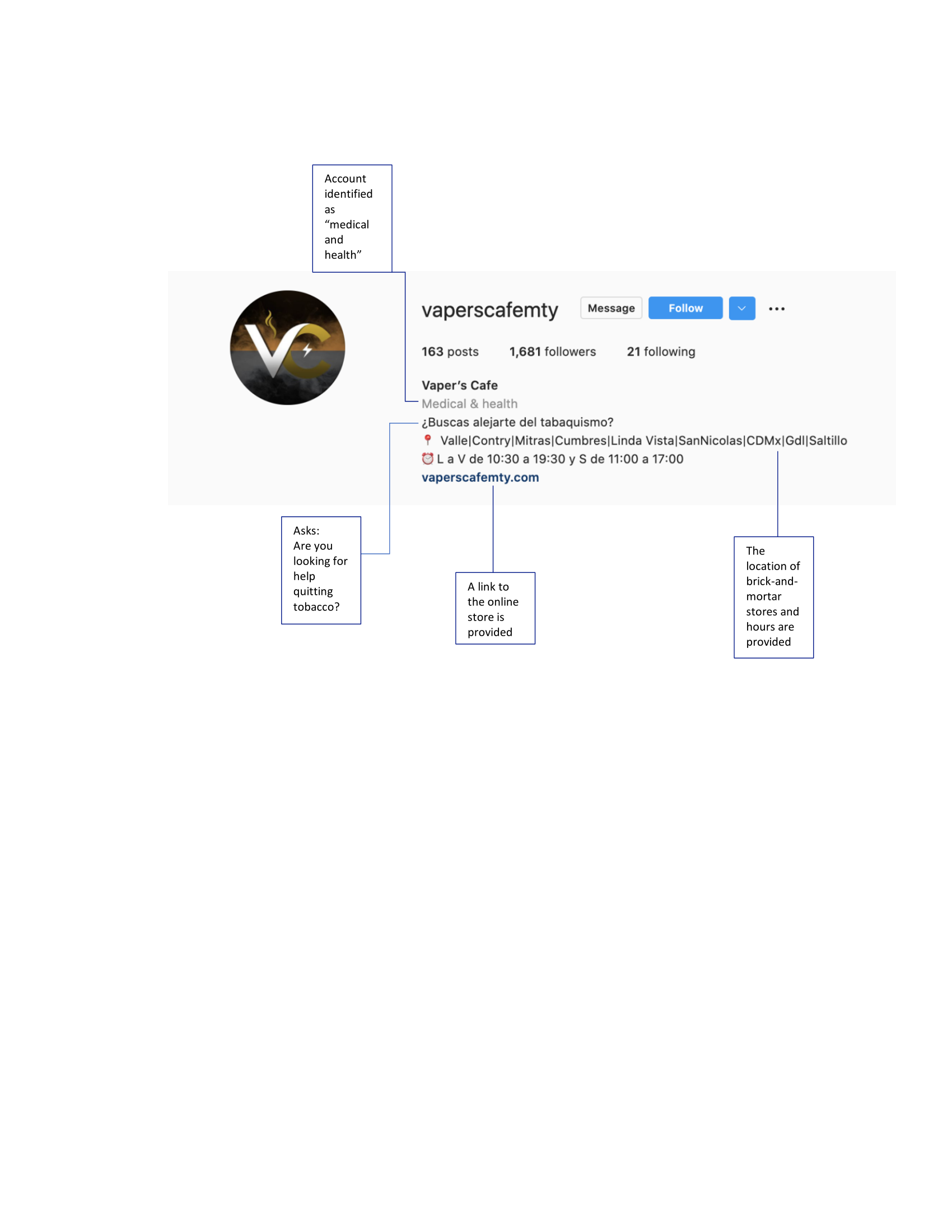

Supplement: Supplementary Image 1 — A HexOhm Mojokerto community meet in Indonesia promoted via the product brands' Instagram page. [file Data_Sheet_1.ZIP › Supplementary Material Presentation/Figures3a-c_Avenues for purchase of e-cigarettes promoted in Instagram posts in India, Indonesia and Mexico.tiff]

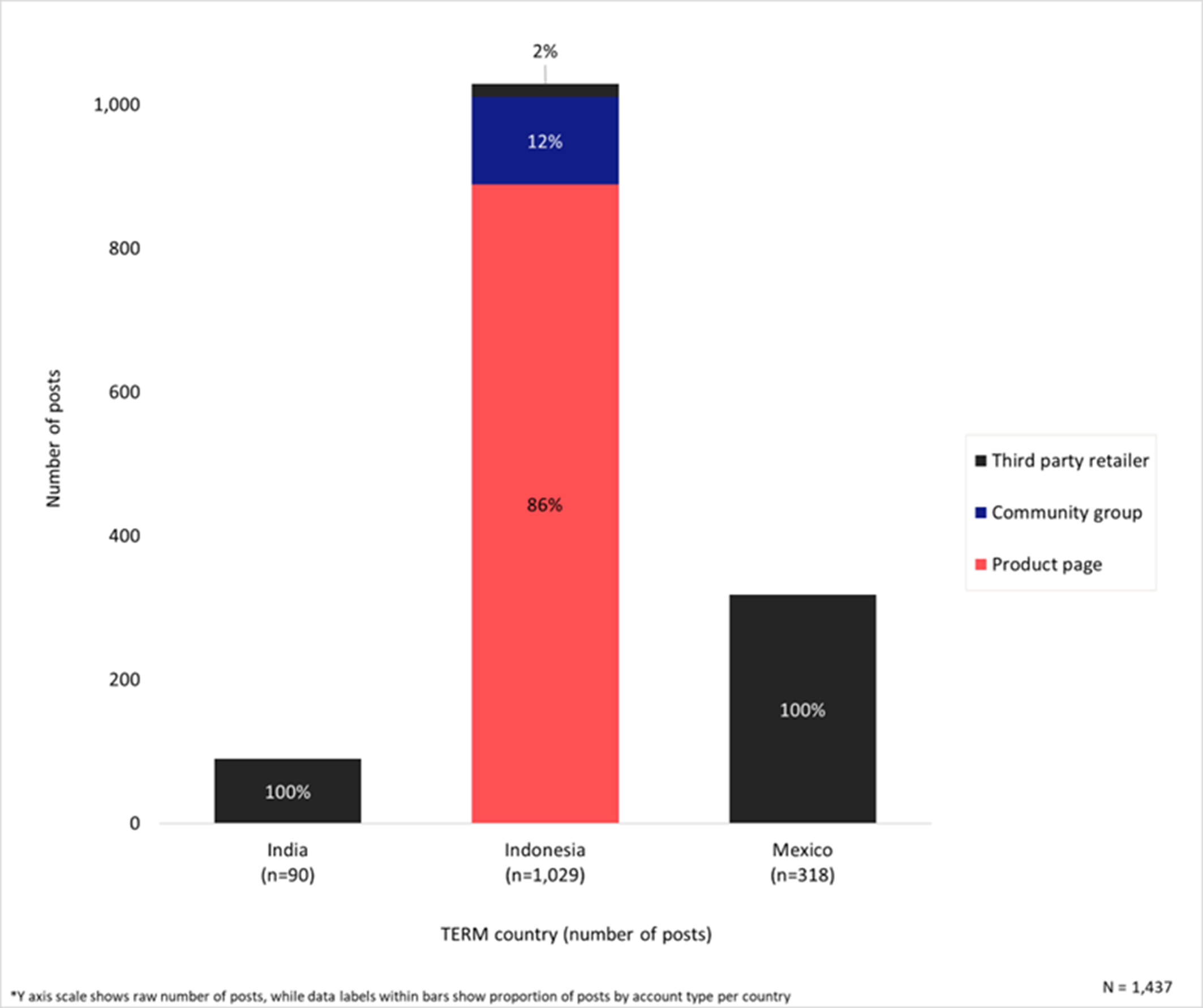

Supplement: Supplementary Image 1 — A HexOhm Mojokerto community meet in Indonesia promoted via the product brands' Instagram page. [file Data_Sheet_1.ZIP › Supplementary Material Presentation/Figure2_Percentage of e-cigarette marketing by account type in India, Indonesia and Mexico.tif]
