# Supplementary material for: A content analysis of e-cigarette marketing on social media: Findings from the Tobacco Enforcement and Reporting Movement (TERM) in India, Indonesia and Mexico
Source: Front Public Health. 2022 Nov 8;10:1012727. doi: 10.3389/fpubh.2022.1012727 (PMC9679495; doi:10.3389/fpubh.2022.1012727)
Supplement: Supplementary file 2 [file Table_1.docx]

| Appendix 1: Codebook for "A content analysis of e-cigarette marketing on social media: Findings from the Tobacco Enforcement and Reporting Movement (TERM) in India, Indonesia and Mexico" | |
| --- | --- |
| (The following is a succinct version of the codebook intended to provide definitions for each variable used in the analysis for this paper) | |
|  | |
| **Appendix Table 1**. Codebook for “A content analysis of e-cigarette marketing on social media: Findings from the Tobacco Enforcement and Reporting Movement (TERM) in India, Indonesia and Mexico” | |
| **Variable** | **Codebook definition** |
| ***Platform*** | We tracked the following e-cigarette marketing on these platforms: |
| Facebook | Captures organic (non-paid ads) posts posted to public Facebook accounts |
| Instagram | Captures organic posts posted to public Instagram accounts |
| News | Captures news stories about e-cigarette companies/brands via keyword searches |
| Twitter | Captures organic tweets posted to public Twitter accounts |
| YouTube | Captures organic videos posted to public YouTube accounts |
| TikTok | Captures organic videos posted to public TikTok accounts |
| ***Media name*** | Name of media/social media account that published the post (what appears after the @ sign for social media) |
| ***Marketing tactic*** | Type of marketing strategy used, which captures the intention of the marketing (to sell products, create brand endearment/loyalty, etc.) |
| Direct marketing | Promotes the sale or use of tobacco and new nicotine and tobacco products in a general way |
| Price promotions | Promotes the sale or use of tobacco products and new nicotine and tobacco products through price discounting (e.g., 10% off) |
| Events, occasions, sponsorships | Pairs the company/brand or tobacco/nicotine product with events, occasions, sponsorships and contests (e.g., product sponsorship of sports events, live concerts, holiday greetings) |
| Corporate social responsibility | Pairs the company/brand or product with socially responsible activities (e.g., water conservation projects, financing vaccination drives) |
| General profile raising | Intended to raise the profile of the brand/company but does not fall into any of the other categories (e.g., brand/company wishing a happy new year or Christmas) |
| ***Message framing*** | Underlying theme around which the whole message of the post is built. |
| Community celebrations and festivals | Posts commemorating a specific event or community celebration. This category also includes posts commemorating birthdays or deaths of famous people including religious figures and politicians. |
| Entertainment | Posts that promote the product as being entertaining and fun. This category also includes posts that use entertainment-related content including memes, funny videos or jokes. |
| Environment eco-awareness | Any post that addresses climate change, conservation, sustainable development goals, or encouraging people to support environmental causes. |
| Glamorization | Posts that associate use of products as aspirational, luxurious or part of an ideal, fashionable lifestyle. |
| Health claims | Posts that present the product as healthier than other products or as being less harmful than conventional tobacco products. |
| Instructional | Posts that instruct viewers how to use a product. This category also includes posts that provide background information on the company. |
| Personal care and wellness | Posts that associate products with relaxation or stress management. This category also includes posts that frame products as being used for social bonding, including forming community around use of the product with like-minded peers |
| Product features | Posts that primarily emphasize the available choices of product flavors and design including device colors, as well as technical specifications of the product. Posts without any text descriptions or keywords that only display the product are also included in this category |
| Social welfare | Posts that showcase activities sponsored or supported by company/brand that are meant to improve their public image. Any posts associating the company with social welfare schemes, livelihood initiatives, women’s empowerment, entrepreneurship, educational scholarships, etc. |
| ***Type of tobacco*** | We tracked the type of tobacco product being marketed (extracting those tagged "e-cigarette" for this study) |
| E-cigarettes | Electronic and/or battery-operated devices designed to deliver an inhaled dose of nicotine and/or other substances, examples of which include “vapes,” “vape pens,” mods, tanks. We also capture marketing for e-liquids. |
| Smoking | A type of tobacco that is burned and the smoke is inhaled, such as cigarettes, cigars, kreteks and bidis |
| Smokeless | A type of tobacco that is sucked or chewed, such as chewing tobacco and betel quid |
| Heated tobacco product | Tobacco products that require the use of an electronic device to heat a stick or pod of compressed tobacco. |
| Other new nicotine products | Products that contain nicotine, such as nicotine pouches |
| **Likes/loves** | Number of likes or loves |
| **Comments/replies** | Number of comments on the post |
| **Shares** | Number of times the post was shared |
| **Engagement** | We tracked the total engagement or the sum total of user activity with the post. The engagement refers to engagement with the published post or video by liking/loving it, commenting/replying to it or sharing it with others |
