# Supplementary material for: A content analysis of e-cigarette marketing on social media: Findings from the Tobacco Enforcement and Reporting Movement (TERM) in India, Indonesia and Mexico
Source: Front Public Health. 2022 Nov 8;10:1012727. doi: 10.3389/fpubh.2022.1012727 (PMC9679495; doi:10.3389/fpubh.2022.1012727)
Supplement: Supplementary file 3 [file Table_2.docx]

**Appendix Table 2. Characteristics of accounts marketing e-cigarette and e-liquid products:**

| Country | Platform | Account name | Account description | Total followers / subscribers (n) | Posts during study period (n) |
| --- | --- | --- | --- | --- | --- |
| **India (N=7)** |  | **Retailers** |  |  |  |
|  | Instagram | vape_wholesaler_india | No classification | 1,110 | 15 |
|  | Instagram | tugboat.india | No classification | 4,724 | 30 |
|  | Instagram | Tugboatvapeindia | No classification | 27 | 25 |
|  | Instagram | vape_bar_india | No classification | 730 | 1 |
|  | Instagram | vapers_stop_india | Product/service | 1,120 |  |
|  | Instagram | indian_vape_shop | Brand | 3,870 | 3 |
|  | Instagram | theindiavapeshop | No classification | 82,900 |  |
| **Indonesia (N=15)** |  | **Retailers** |  |  |  |
|  | Instagram | roots_vape | No classification | 3,750 | 3 |
|  | YouTube | Panda Vapestore | No classification | 605,000 | 8 |
|  |  | **Brand accounts** |  |  |  |
|  | Instagram | geekvape.indonesia | Product/service | 53,800 | 52 |
|  | Instagram | Vaporcaksuroboyo | Shopping and retail | 20,500 | 7 |
|  | Instagram | smok_indonesia | Product/service | 36,400 | 84 |
|  | Instagram | upods_id | Community | 72,400 | 96 |
|  | Instagram | voopoo_indonesia | Electronics | 54,300 | 179 |
|  | Instagram | Uwell.indonesia | Product/service | 10,100 | 10 |
|  |  |  |  |  |  |
|  | Facebook | GeekVape.Indonesia | Product service | 9,240 | 172 |
|  | Facebook | SMOK_Indonesia | Electronics | 2,800 | 95 |
|  | Facebook | voopooindonesia | Electronics | 4,080 | 190 |
|  | Facebook | Uwell Indonesia | Electronics | 770 | 6 |
|  |  |  |  |  |  |
|  | Twitter | GeekvapeTech | No classification | 17,400 | 5 |
|  |  | **Community Groups** |  |  |  |
|  | Instagram | hexohmindonesia | Community organization | 72,700 | 64 |
|  | Facebook | hexohmindonesia | Community organization | 12,770 | 58 |
| **Mexico (N= 13)** |  | **Retailers** |  |  |  |
|  | Instagram | ecig_depot | Personal blog | 2,630 | 19 |
|  | Instagram | kapitalsmokeandvapor | No classification | 3,020 | 72 |
|  | Instagram | Ecigmexvapeshop | E-cigarette store | 2,600 | 5 |
|  | Instagram | Vaperscafemty | Medical & health | 1,680 | 1 |
|  | Instagram | centralsmokeandvape | No classification | 1,320 | 1 |
|  |  |  |  |  |  |
|  | Facebook | ecig.mx | Shopping & retail | 5,433 | 58 |
|  | Facebook | Vapor a la Mexicana MX | E-cigarette store | 11,000 | 16 |
|  | Facebook | lavaperiamx | E-cigarette store | 7,430 | 31 |
|  | Facebook | kapitalsmokeandvapor | Product/service | 5,592 | 62 |
|  | Facebook | thehighclubsmokeshop | Accessories | 42,050 | 6 |
|  | Facebook | Vapers Cafe Monterrey | Product/service | 10,838 | 3 |
|  |  |  |  |  |  |
|  | TikTok | La soberbia store | No classification | 1,170 | 1 |
|  | TikTok | ecig.mx | No classification | 2,270 | 2 |
