# Supplementary material for: A content analysis of e-cigarette marketing on social media: Findings from the Tobacco Enforcement and Reporting Movement (TERM) in India, Indonesia and Mexico
Source: Front Public Health. 2022 Nov 8;10:1012727. doi: 10.3389/fpubh.2022.1012727 (PMC9679495; doi:10.3389/fpubh.2022.1012727)
Supplement: Supplementary file 4 [file Table_3.docx]

**Appendix Table 3: Examples of keyword-based Boolean searches**

| Country | Topic | Keywords |
| --- | --- | --- |
| India | SMOK e-cigarette brand | (smok" OR "#smok" OR "smok tech" OR "#smoktech" OR "smok technology" OR "#smoktechnology" OR "#SMOKSCAR" OR "#SMOKNordX" OR "#SMOKRIGEL" OR "#SCAR18" OR "#SMOKNFIX" OR "#SCARseries" OR "#SMOKRPM160" OR "#SMOKRPM2" OR "#SCARP5" OR "#SCARP3" OR "#SMOKNOVOX" OR "#RPM2" OR "#smokrpm" OR "#novoXkit" OR "#RPM160" OR "@SMOKTECHLOGY" OR "#TFV9Tank" OR "#NORDKIT" OR "#nexMESHPod" OR "#SPECIES" OR "#SMOKFAM" OR "#RPM40" OR "#RPMlite" OR "#SMOKAlike" OR "#smokshow" OR "#smokvape" OR "#SMOKscarP3" OR "#ScarP3" OR "समोक" OR "#समोक" OR "समोक तेच" OR "#समोकतेच" OR "समोक तेच्नोलोग्य" OR "#समोकतेच्नोलोग्य") AND (marketing OR "#marketing" OR promotion OR "#promotion" OR event OR "#event" OR advertise OR advertisement OR "#advertise" OR "#advertisement" OR "promotion" OR "#promotions" OR advertising OR "#advertising" OR "buy best" OR "win" OR "sale" OR "discount" OR "#buy" OR "#win" OR "#sale" OR "#discount" OR sponsor OR "#sponsor" OR "#sponsorship" OR "sponsorship" OR music OR festivals OR concerts OR webinars OR literarure OR art OR "sports events" OR seminar OR Conferences OR "trade shows" OR workshops OR parties OR Reunions OR "Corporate Events" OR "Social Events" OR "Award Function" OR "मार्केटिंग " OR "सर्वश्रेष्ठ खरीदें" OR "जीत" OR "बिक्री" OR "छूट" OR "# ब्यू" OR "#विन" OR "#पैमाने" OR "#छूट" OR प्रायोजक OR "#प्रायोजक" OR "#पूजा" OR "प्रायोजन" OR संगीत OR त्यौहार OR "संगीत समारोह" OR वेबिनार OR साहित्यिक OR कला OR "खेल आयोजन" OR संगोष्ठी OR सम्मेलन OR "व्यापार शो" OR कार्यशालाएं OR पार्टी OR पुनर्मिलन OR "कॉर्पोरेट इवेंट्स" OR "सामाजिक कार्यक्रम" OR "पुरस्कार समारोह")" |
|  | VOOPOO e-cigarette brand | (voopoo" OR "#voopoo" OR "voopoo tech" OR "#voopootech" OR "#voopootechnology" OR "voopoo technology" OR "#voopoovthrupro" OR "#voopoovsuit" OR "#VoopooArgus" OR "#VoopooArgusAir" OR "#VoopooArgusGT" OR "#voopoodrag" OR "#voopooDRAG" OR "#voopooARGUS" OR "#Vthrupro" OR "#Vsuit" OR "#Vthru" OR "#voopoomoment" OR "#voopoovsuit" OR "#voopoovthrupro" OR "#vooppovinciair" OR "#voopoodragx" OR "#voopoovincix" OR "#voopoodragminipt" OR "#voopooargusgt" OR "वॊपॊ" OR "#वॊपॊ" OR "वॊपॊ तेच" OR "#वॊपॊतेच" OR "वॊपॊ तेच्नोलोग्य" OR "#वॊपॊतेच्नोलोग्य") AND (marketing OR "#marketing" OR promotion OR "#promotion" OR event OR "#event" OR advertise OR advertisement OR "#advertise" OR "#advertisement" OR "promotion" OR "#promotions" OR advertising OR "#advertising" OR "buy best" OR "win" OR "sale" OR "discount" OR "#buy" OR "#win" OR "#sale" OR "#discount" OR sponsor OR "#sponsor" OR "#sponsorship" OR "sponsorship" OR music OR festivals OR concerts OR webinars OR literarure OR art OR "sports events" OR seminar OR Conferences OR "trade shows" OR workshops OR parties OR Reunions OR "Corporate Events" OR "Social Events" OR "Award Function" OR "मार्केटिंग " OR "सर्वश्रेष्ठ खरीदें" OR "जीत" OR "बिक्री" OR "छूट" OR "# ब्यू" OR "#विन" OR "#पैमाने" OR "#छूट" OR प्रायोजक OR "#प्रायोजक" OR "#पूजा" OR "प्रायोजन" OR संगीत OR त्यौहार OR "संगीत समारोह" OR वेबिनार OR साहित्यिक OR कला OR "खेल आयोजन" OR संगोष्ठी OR सम्मेलन OR "व्यापार शो" OR कार्यशालाएं OR पार्टी OR पुनर्मिलन OR "कॉर्पोरेट इवेंट्स" OR "सामाजिक कार्यक्रम" OR "पुरस्कार समारोह")" |
| Indonesia | GeekVape e-cigarette brand | (#geekvapeindonesia" OR "#geekvapezeus" OR "#geekvapenova" OR (("geekvape" OR "#geekvape" OR "#geekvapes" OR "geekvapes" OR "geekvapeindonesia") AND (vape OR "#vape" OR vapes OR "#vapes" OR "#vapeindonesia" OR "#vapelife" OR "#vapenation" OR "#vapecommunity" OR "#vapersindonesia" OR "#vapeindonesian" OR "#vapeindo" OR "#vapejakarta" OR "#vapeworld" OR "#vapedaily" OR "#vapefamous" OR "#vapeallday" OR "#vapeaddict")))" |
|  | E-cigarette | (((electronic smoking devices" OR "E Cigarettes" OR "E-Cigarettes" OR "#ecigarette" OR "#ecigarettes" OR "#ecigar" OR "#ecigars" OR "#ecigarete" OR "#ecigarrete" OR "#ecigaretteindonesia" OR "alat rokok elektonik" OR "Rokok elektronik" OR "#rokokelektronik" OR Vaping OR Vape OR "#Vape" OR "#Vaping" OR "#vapelifeindonesia" OR hookah OR "#hookah") AND (marketing OR "#marketing" OR promotion OR "#promotion" OR event OR "#event" OR advertise OR advertisement OR "#advertise" OR "#advertisement" OR "promotion" OR "#promotions" OR advertising OR "#advertising" OR "buy best" OR "win" OR "sale" OR "discount" OR "#buy" OR "#win" OR "#sale" OR "#discount" OR sponsor OR "#sponsor" OR "#sponsorship" OR "sponsorship" OR pemasaran OR "#pemasaran" OR promosi OR "#promosi" OR iklan OR #iklan OR musik OR konser OR sastra OR "event olahraga" OR konferensi)) OR "Vaporous Marketing")" |
| Mexico | Vaporesso e-cigarette brand | (vaporesso OR #vaporesso" OR "vaporesso e cigarette" OR "vaporesso e- cig" OR "vaporesso vape" OR "#vaporesso_ecig" OR "#vaporesso_ecig_mod" OR "#vaporessoecig" OR "#vaporessogen" OR "#vaporessogenkit" OR "#vaporessoecig" OR "#vaporessogens" OR "#vaporessoshow" OR "#vaporessoauroraplay" OR "#vaporessoclick" OR "#vaporessopodstick" OR "#vaporessogtxgo80" OR "#vaporessogtxgo40" OR "#vaporessogtxgo" OR "#gtxgo40" OR "#gtxgo40kit" OR "#vaporessogtxcoil")" |
|  | E-cigarette | (((electronic smoking devices" OR " E Cigarettes" OR " E-Cigarettes" OR "#ecigarette" OR "#ecigarettes" OR "#ecigar" OR "#ecigars" OR "#ecigarete" OR "#ecigarrete" OR "#ecigarettemexico" OR Vaping OR Vape OR "#Vape" OR "#Vaping" OR "#vapelifeindia" OR hookah OR "#hookah" OR "cigarros electronicos" OR "#e-cig" OR "#ecig" OR Vapear OR "#Vapear" OR "Vapeo" OR "#Vapeo" OR "#vapelifemexico" OR "#elvapeosalvomivida" OR "#elvapeosalvavida") AND (marketing OR "#marketing" OR promotion OR "#promotion" OR event OR "#event" OR advertise OR advertisement OR "#advertise" OR "#advertisement" OR "promotion" OR "#promotions" OR advertising OR "#advertising" OR "buy best" OR "win" OR "sale" OR "discount" OR "#buy" OR "#win" OR "#sale" OR "#discount" OR sponsor OR "#sponsor" OR "#sponsorship" OR "sponsorship" OR promocion OR "#promocion" OR evento OR "#evento" OR anuncio OR "anuncio publicitario" OR "#ad" OR "#anuncio" OR "promocion" OR "#promociones" OR "gana" OR "venta" OR "descuento" OR "#compra" OR "#compra" OR patrocinio OR "#patrocionio" OR "#patrocinado" OR "patrocinado")) OR "Vaporous Marketing")" |
